# Supplementary figures and images for: Improving Syrian refugees’ knowledge of medications and adherence following a randomized control trial assessing the effect of a medication management review service
Source: PLoS One. 2022 Oct 14;17(10):e0276304. doi: 10.1371/journal.pone.0276304 (PMC9565448; doi:10.1371/journal.pone.0276304)

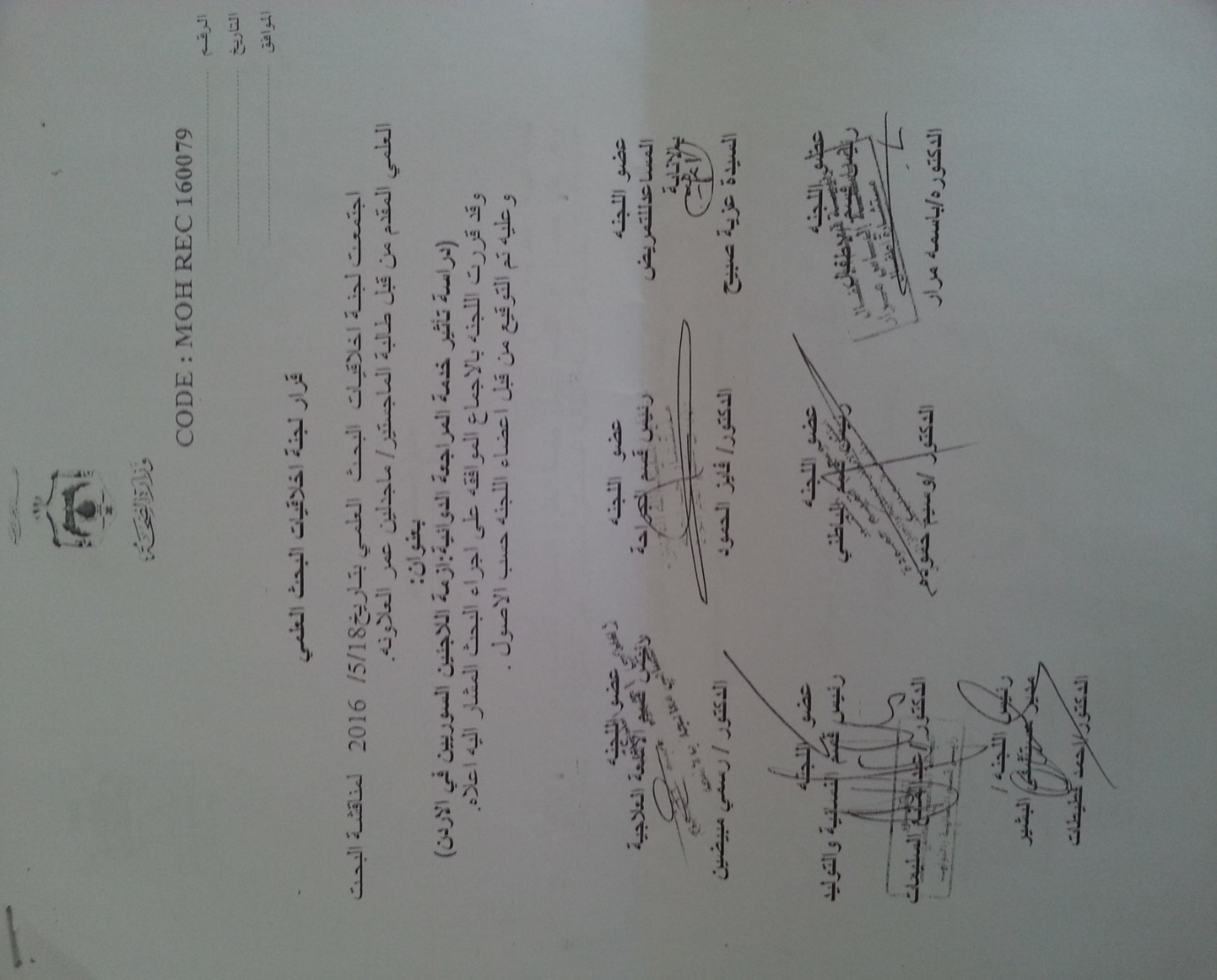

Supplement: S1 File — (DOCX) [file pone.0276304.s002.docx]
