# Supplementary material for: Improving Syrian refugees’ knowledge of medications and adherence following a randomized control trial assessing the effect of a medication management review service
Source: PLoS One. 2022 Oct 14;17(10):e0276304. doi: 10.1371/journal.pone.0276304 (PMC9565448; doi:10.1371/journal.pone.0276304)
Supplement: S2 File — (DOCX) [file pone.0276304.s003.docx]

Syrian Refugees Crises in Jordan: The Impact of Medication Management Review Service

**Introduction**

Prior research defined the Medication Errors (MEs) as a "failure in the treatment process that leads to, or has the potential to lead to, harm to the patient" (Aronson, 2009). MEs are gaining increasing attention in the recent years due to being the eighth cause of death in the USA according to the Institute of Medicine [IOM] (IOM, 1999). It has been documented that as many as 200,000 people subsided per year as a result of MEs in the USA (Stafford *et al*., 2009). It is apparent from the MEs definition that they are a multidisciplinary issue and their causes could be multifactorial, and therefore a strategic and multidiscilinary method to mitigate the occurrence of these MEs has to be applied (Brulhart & Wermeille, 2011; Cohen, 2007; O'Shea, 1999).

The MEs and the resultant Drug Related Problems (DRPs) are considered a serious problem that charges the medical sector a huge financial cost in most countries (Kothari *et al*., 2010; Pinilla *et al*., 2006). According to the Pharmaceutical Care Network in Europe, DRP is defined as ‘an event or circumstance involving drug therapy that actually or potentially interferes with desired health outcomes’ .Research data estimated that more than 1.5 million preventable DRPs occur each year in the USA, accounting for an extra load of $177 billion in term morbidity and mortality (American Pharmacists Association, 2008). In a wider scale, literature review for DRPs has shown that 28% of all emergency department visits were drug related, of which 70% were avoidable (Patel & Zed, 2002).

One of the best ways to save human lives, optimize health care and save the financial losses resulting from MEs and consequently DRPs is the so-called medication management review (MMR) service. MMR service is an application of Pharmaceutical Care (PC), where it is defined as ''a distinct service or group of services that optimize clinical outcomes for each patient to ensure the appropriateness, effectiveness, safety for each patient‘s medication(s), along with the ability of the patient to take their medication(s) as should be'' (American Pharmacists Association (APhA), 2004).

Several studies have manifested the effect of PC and MMR on improving patients' outcomes. The positive impact of pharmacist follow-ups on primary outcome of the patient's health have been indicated by several Australian and British studies (Gilbert *et al.*, 2002; Bergheim *et al.*, 2008). In national context, the positive impact of home-based medication review for patients with chronic conditions has been proven (Basheti *et al*., 2016).

To apply the MMR service that reduces the Treatment Related Problems (TRPs), drug-related visits to the emergency department and the subsequent morbidity and mortality, an environment of cooperation must exist between pharmacists and physicians (Zed, 2002).

Pharmaceutical care services (PCSs) that conducted by accredited pharmacist around the world included different patterns of Medication Reviews, such as the Medication Therapy Management (MTM) in the USA (Oliveira *et al*., 2010) and the Home Medication Review (HMR) in Australia (Basheti *et al*., 2013). In United Kingdom, medicines use reviews’ (MURs) program exists as a part of England and Wales NHS contracts , and similar service exists in Scotland which is the Chronic Medication Service (CMS) as a part of the core community pharmacy contracts (Blenkinsopp *et al*., 2012). This advancement in health care in the developed countries is much different from what has been found in the developing countries (Rao *et al*., 2007).

Although the MMR services have proved to be very effective as it shown in the developed countries, the situation in the developing countries is different. Despite the multiplicity of reasons and the importance of having such services in developing countries, the current situation does not reflect that. Self-medication behavior, excessive dosing, financially related non-adherence, considerable use of herbs, short time of physician-patient consultation and the suboptimal role played by pharmacists are among the reasons that call for such interventions (Yussuf and Tayo, 2011; Khan *et al*., 2012; Jassim 2010). In Jordan, the need for MMR is high as many of the pre mentioned issues exist (Basheti *et al*., 2013; Wazaify *et al*., 2008). When the need arises and for the reasons previously mentioned, Basheti *et al*. (2016) conducted a study to evaluate the impact of home-based medication review for patients with chronic conditions in Jordan. The study concluded that the MMR service has efficiently decreases the total number of TRPs and has improved the patient self-report adherence (Basheti *et al*., 2016).

Refugees all over the world are facing several health-related problems (Burgess, 2004). In general most of those refugees are not gaining proper medical care (Pfortmueller *et al*., 2016). Since the Syrian revolution started in March 2011 right across the Jordanian border, thousands of Syrians have crossed into northern Jordan. Syrian refugees become a significant part of Jordanian society, which counting for tenth of Jordanian population according to the latest statistics of United Nations High Commissioner for Refugees (UNHCR)^^[[1]](#footnote-1)^^. To cope with this high number of refugees, five camps are established now in Jordan and another is being planned in the future (Al-Kilani, 2014; Basheti *et al*., 2015). However, most of Syrian refugees are living outside the camps where most of their health and humanitarian needs cannot be sufficiently addressed (Murshidi *et al*., 2013). A survey conducted by five reliable international and national authorities, found that the existence of one or more of the chronic diseases among Syrian refugees household members in Jordan was accounted for 43.4%^[[2]](#footnote-2)^. This increasing number of refugees with multiple chronic conditions is in need for urgent medical care.

Pharmacist during the last two decades became an important and efficient member of the healthcare team and contributed substantially to the wellbeing of patients in the different health care settings. Pharmacists have proved to be effective in delivering pharmaceutical care and managing the chronic diseases. Our humanitarian and medical role as pharmacists requires us to make an urgent intervention for this denomination of Jordanian populations. The MMR service would be an ideal service to deliver to the Syrian refugees in Jordan for the many benefits it showed with other population. To conclude, MMR in Jordan involving the Syrian refugees have to be one of the essential issues to be tackled in the pharmaceutical research at this stage. This research is going to address the impact of identifying and resolving TRPs by MMR service on many different aspects of the Syrian refugees' health outcomes.

**The Aim of the Research:**

The primary aim of this study is to explore the impact of the MMR service on refugee's health outcomes (reducing the total number of the identified TRPs), Quality of Life (QoL), the self-reported adherence, knowledge about medication and on the improvement on the scale of anxiety.

Secondary aim is to assess the acceptability rate of physicians to MMR service delivered to the Syrian refugees and to measure the perspective of these refugees to the MMR service delivered to them. In addition to the mentioned aims, the type and frequency of TRPs found in this population is going to be addressed.

**Study objectives:**

This study sheds light on the following objectives:

1-What is the eventual impact of applying MMR service on decreasing the total number of TRPs identified among study refugees for intervention compared to control group?

2-What is the eventual impact of applying MMR service on the study refugees' Quality of Life (QoL) for intervention compared to control group?

3-What is the eventual impact of applying MMR service on the study refugees' self-reported adherence for intervention compared to control group?

4-What is the eventual impact of MMR service on improving the scale of anxiety for the study refugees for intervention compared to control group?

5-How much will the acceptability rate of the physicians’ acceptance to the MMR service be?

6-What is the physicians' approval rate for the pharmacist’s recommendations regarding the identified TRPs?

7- What are the common types of TRPs identified among the study refugees?

8-What are the frequencies of TRPs identified among the study refugees?

9-What is the intervention group’s perspective of the MMR service?

**Research Hypothesis**

*Null hypothesis*: The implementation of the MMR service for adult Syrian refugees with chronic conditions in Jordan will have no impact on the number of identified TRPs, anxiety scale, self-reported adherence, patients’ knowledge about their medication and quality of life in comparison to patients that will not receive this service.

*Alternative hypothesis:* The implementation of the MMR service for adult Syrian refugees with chronic conditions in Jordan will have significant impact on the number of identified TRPs, anxiety scale, self-reported adherence, patients’ knowledge about their medication and quality of life in comparison to patients that will not receive this service.

**Literature Review and Theoretical framework**

**1. Medication Errors:**

Medication is clearly the most common form of medical intervention (Guide to medication review, 2002). Medication Errors (MEs) can be defined as a "failure in the treatment process that leads to, or has the potential to lead to, harm to the patient" (Aronson, 2009). The Institution of medicine (IOM) revealed that among all medical errors, MEs are the most prevalent (Institute of Medicine, 1999). It is evident that medication errors contribute in morbidity and death (Leape, 1994; Kohn *et al*., 1999; Phillips & Bredder, 2002). Ferner & Aronson (2006) classified the MEs into mistake- related MEs (knowledge-based or rule-based) and skills-related MEs (action-based or technical errors or memory-based). MEs are frequent in many clinical settings, can occur at any point in the medication use process and are much more common than adverse drug events (ADEs) (Morimoto *et al*., 2004; Cheragi *et al*., 2013). United stated FDA and world health organization defined the ADE as “any untoward medical occurrence that may present during treatment with a pharmaceutical product but which does not necessarily have a causal relationship with this treatment” (Nebeker *el al*., 2004). Morimoto *et al*. (2004) found that about a third to half of ADEs is typically related to MEs. Therefore, to confirm the safety in medication use, health professional must consider the ''five rights'' of the drug use: right patient, right drug, right dose, right rout and right time (Cohen, 2007). Any deviation from these rights may cause *''medication error''*. Many studies revealed that the MEs are common and not new or emerging problem (Cobb, 1986; Elliott *et al*., 2016). In 1992, this idea was confirmed through a study conducted in Boston among an urban tertiary care hospital; which counted for an average of 5.3 medication errors/100 order over a 51-day study (Bates *et al*., 1995).

Previous research showed that there are many causes for MEs. Cohen, (2007) listed these causes as the following: Lack of knowledge of the drug, lack of information about the patient, violation of health care rules, slips and memory lapses, transcription errors, faulty interactions with patients care services, faulty dose checking, inadequate monitoring, lack of standardization and preparation errors (Cohen, 2007). Matthew *et al*. (2012) explained that the deficiency in primary medical record of the patients is one of the crucial causes of the MEs. Therefore, the MEs causes are multi factorial, cutting across many lines of responsibilities (Cohen, 2007).

**2. Cost of Medication Errors:**

The literature showed that the cost of improper drug use is huge for both patients and healthcare systems in particular in countries with limited resource (Otoom and Sequeira, 2005). According to the Pharmaceutical Care Network in Europe, Drug Related Problem (DRP) is defined as ‘an event or circumstance involving drug therapy that actually or potentially interferes with desired health outcomes^[[3]](#footnote-3)^. DRPs resulting from MEs are an important cause of emergency department visits and subsequent resource use (Patel, 2002). In addition, 140,000 hospital admissions have resulted from DRPs in Australia, and between 32% and 69% of those admissions were suggested to be preventable (Roughead, 1999; Roughead *et al*., 2004). The estimated cost of DRPs in the USA is high and reached $US 177 billion on 2000 (Ernst and Grizzle, 2001; Woodward *et al*., 2010). Research data estimated that more than 1.5 million preventable ADEs occur each year in the United States, accounting for an extra load of $177 billion in term of DRPs' morbidity and mortality (American Pharmacists Association, 2008). The DRPs has been proven to be accountable for $US100 billion in direct and indirect costs (Pellegrino *et al*., 2009). Another study in Europe found that the average cost per case for the total number of patients attending during 2001 was €1,435 (Pinilla *et al*., 2006) due to MEs. In conclusion, MEs are a costly health-system issue that requires immediate attention and resolution.

**3. Treatment Related Problems**

The term DRP is widely used in the literature and is approved by major clinical areas across the world .However, it has been chosen to use the term ‘‘treatment-related problems’ (TRP) in this study to expand the area of PC practice, as the term DRP, especially when translated into the Arabic language, might limit the range of ‘pharmaceutical care’ to ‘drug related care’ (AbuRuz *et al*., 2006; AbuRuz *et al*., 2011). TRP can be defined as “an event or circumstance involving patient treatment that actually or potentially interferes with an optimum outcome for a specific patient” (AbuRuz, *et al*., 2006, Aburuz, *et al*., 2011).

It has been demonstrated in a previous longitudinal German study conducted between 2003 and 2007 that more than 5% of Hospital visits are either caused or complicated by TRPs (Stausberg and Hasford, 2011). TRPs can be categorized into: indication related problems, drug related problem, safety related problem, patient related problem, efficacy related problem, knowledge related problems and adherence related problems (AbuRuz *et al*., 2011).

Basheti *et al*. (2013) study revealed that the identified TRPs in Jordanian outpatients with chronic diseases visiting community pharmacies are high (Basheti *et al*., 2013). AbuRuz *et al*. (2011) conducted a study in the largest general hospital in Jordan and found high prevalence of TRPs within hospitalized internal medicine patients. Among all TRPs identified in the country, DRPs was the highest (AbuRuz *et al*., 2011).

**4. Pharmaceutical Care:**

During the last decade, many studies announced that the Pharmaceutical Care (PC) and the community pharmacy interventions mostly lead to desirable clinical and financial outcomes (Abughosh *et al*., 2016).

Hepler & Strand (1990) have defined the PC as ‘‘the responsible provision of drug therapy for the purpose of achieving definite outcomes that improve a patient’s quality of life, which is a widely accepted definition for the PC. The previous definition described three major functions of PC which included: identifying potential and actual DRPs, resolving actual DRPs and preventing potential DRPs (American Society of Health-System Pharmacists, 2015). Pharmaceutical care has improved the use of medications, patients’ awareness about medications, adherence to medications, the physician's prescribing and the overall patient’s quality of life, which is the fundamental goal of providing pharmaceutical care (AbuRuz *et al*., 2006; Mahdikhani & Dabaghzadeh, 2016**).** It has been explained that identifying, resolving and preventing the TRPs are among the crucial responsibilities of the pharmacists when supplying PC (Viktil & Blix, 2008**).** The importance of MMR are shown clearly due to the fact that 50% of medicines are not being taken by patients as prescribed (Department of Health, 2001; Royal Pharmaceutical Society of Great Britain). (Yussuf & Tayo, 2011) reported that the enforcement of the potentially care-enhancing role of the clinical pharmacists is the first step towards improving the quality of medication use in all developing countries.

However, Clinical pharmacy and pharmaceutical care are prospering more in the developed countries than with developing countries such as Jordan (AbuRuz *et al*., 2006). Despite that the physicians in Jordan started to accept the role of the clinical pharmacist among the health care team, and despite the high acceptance rate of pharmacists’ recommendations by the physicians, (AbuRuz *et al*., 2011), data showed that the level of pharmaceutical care activities is still limited in the country (AbuRuz *et al*., 2012). To our advantage, Jordanian pharmacists in general have very good perspective toward pharmaceutical care and 90% of lay people are fully encouraging the concept of PC (AbuRuz *et al*., 2012).

**5. MMR Service in Developing Countries:**

Medication-related malpractices are common all over the world, and it is not distinctive to developing countries, where both developed and underdeveloped countries are suffering from consequences of inappropriate use of medications in their health care facilities (Otoom and Sequeira, 2005; Jassim, 2010; ALBashtawy *et al*., 2015).

The self-medication behavior as one form of medication malpractices has been studied in many developing countries. Many studies revealed that the unauthorized self-medication with herbal remedies or with prescribed medicines was the major cause of irrational use of medicines (Eltayeb and Matowe, 2005; Fakeye *et al*., 2009). This is consistent with the Nigerian study conducted among outpatients diagnosed with chronic diseases, aiming to evaluate the prevalence of this behavior (Yussuf and Tayo, 2011). In this study, after a full medication use review carried out by skilled pharmacists, results showed that the prevalence of self-medication was about 39.3% (Yussuf and Tayo, 2011). Moreover, it has been confirmed that the self-medication with antimicrobial was very high among Iraqi people (Jassim, 2010). It has been reported that the use of herbs as a source of medicines is common in most areas of the Mediterranean (Wazaify *et al*., 2008).

In Jordan, it has been demonstrated that the use of prescribed and OTC medications to manage diseases is widespread among the population (Wazaify *et al*., 2008). ALBashtawy el al. (2015) assured that the prevalence of self-medication problem among the school students in Jordan was very high. Other study confirmed that the high prevalence of self-medication with prescription-only drugs, in addition to uncontrolled abuse of OTCs drugs are very important factors causing the high prevalence of DRPs in Jordan (Basheti *et al*., 2013).

Poor adherence to the prescribed medicines as a form of malpractices is common in both developed and underdeveloped countries, with less than 50% of patients remaining adherent after 12 months of starting therapy for chronic conditions (Pellegrino *et al*., 2009). The non-adherence has been proven to be accountable for 125,000 deaths per year, 10% of all hospitalizations, 23% of nursing home admissions in the USA (Pellegrino *et al*., 2009). The likely negative impact of non-adherence to drug therapy on increasing morbidity and mortality cases among patients with chronic diseases is well documented (Brummel & Carlson, 2016).

Many studies suggest that the economic problems in the developing countries are the most common factor leading to non-adherence. Yussuf and Tayo (2011) explained that the Financial difficulties facing the most population in resource-limited countries who live below poverty line, resulting in patients going on drug holidays due to difficulties associated with the purchase of so many medications prescribed for them (Yussuf and Tayo, 2011).

A wide study in Saudi Arabia conducted on patients with diabetes found that non-compliance on prescribed medicines is very high, which had a very negative effect on the patients ([Khan](http://www.jfcmonline.com/searchresult.asp?search=&author=Ataur+R+Khan&journal=Y&but_search=Search&entries=10&pg=1&s=0) *et al*., 2012). Basheti *et al*. (2016) conducted a cross sectional study on patient with chronic conditions in Jordan, aiming to identify the factors may negatively affect the adherence of patients. The study found that about one half (46.1%) of patients were non-adherent (Basheti *et al*., 2016).

The non-revelation of self-medication and non-adherence during physicians' visits has made the problem more dominant (Yussuf & Tayo, 2011). This finding seems to reflect insufficient physician-patient interaction, short physician-patient contact time and lack of privacy during clinic visits (Yussuf & Tayo, 2011; Piette *et al*., 2005). This has been confirmed by Otoom & Sequeira (2006) study which showed that both the mean time spent on physician-patient consultations (3.9±3.5 min) and mean pharmacy dispensing time (28.8 ±23.7 s) were short (Otoom & Sequeira, 2006). In addition, the suboptimal use of medication by outpatients and the suboptimal role played by pharmacists have been proven by many studies conducted in Jordan (Basheti *et al*., 2013).

Special services that have the potential to resolve these vital issues are evidently needed in the country (Basheti *et al*., 2013). The MMR service is a specialized service that delivers patient care, providing all the needed time to identify and resolve any TRPs. The idea of Pharmaceutical care services provided by clinical pharmacists is very well accepted by patients in the Arabic world including Iraq, United Arab Emirates and Jordan (Basheti *et al*., 2014). This has been assured by patient satisfaction survey conducted in Jordan showing that 95.3% of respondents agreeing with the Home Medication Management Review (HMMR) service have improved their overall health and well-being (Basheti *et al*., 2013).

**6. MMR around the World:**

It is promising to know that MMR have effectively produced very useful and important information about patients and TRPs in various countries and in different populations which helped optimize patients’ therapy (Oliveira *et al*., 2010). MMR has been established over the last decade among many countries including Australia, UK, USA and Sweden (Castelino *et al*., 2011).

In Australia, accredited pharmacists perform medication reviews for patients at their homes, called “Home Medication Management Review” (HMMR). Any information related to patient history, current medication or any other issues is gathered and interpreted aiming to identify and resolve TRPs; after that, suitable recommendations regarding these TRPs are delivered to the physicians (Stafford *et al*., 2009). This HMMR aims to increase patients benefit from their therapy and prevent TRPs (Stafford *et al*., 2009).

Similar situation has been found in Sweden; where Westerlund & Marklund (2009) conducted the MMR service by pharmacists from 89 Swedish pharmacies with the aim to identify, classify and respond to TRPs for 13 different patient groups. The results showed that 358 cases (68%) had a significant improvement concerning the therapeutic effect in the patients and 172 cases (32%) had prevented or relieved DRPs (Westerlund & Marklund, 2009). From this study, it has been demonstrated that the extrapolated financial saving can reach €358 million yearly in Sweden as a result of pharmaceutical care application and this is surprisingly 37 times the expected pharmacists personnel costs for identifying and responding to the DRPs (Westerlund & Marklund, 2009).

As for the USA, the service called “Medication Therapy Management” (MTM) which has been defined as “a distinct service or group of services that optimize therapeutic outcomes for individual patients [that] are independent of, but can occur in conjunction with, the provision of a drug product” (McGivney *et al*., 2007). This service aims to improve the improper use of medicines, increase patients’ adherence with their therapy, reduce the ADEs associated with improper medication use and reduce the need for other medical services, which in turn would reduce the cost on health care systems (McGivney *et al*., 2007 ). Oliveira *et al*. (2010) confirmed that the MTM service conducted over 10 years in the USA resulted in clinical outcome improvements and huge cost savings.

When it comes to the UK, the medication review is now widely considered as a cornerstone of medicines management (NHS Cumbria Medicines Management Team, UK, 2011). In the UK it has been termed ‘Medicines Use Reviews’ (MURs) service and defined as “a structured, critical examination of a patient’s medicines with the objective of reaching an agreement with the patient about treatment, optimizing the impact of medicines, minimizing the number of medication related problems and reducing waste” (A guide to medication review,2002,UK). NHS medicines management team declared that supporting and following patients in taking their medications properly by face to face interviews, which is a vital part of MURs service, is crucial for improving patient safety, health outcomes and patient's satisfaction with clinical care (NHS Cumbria Medicines Management Team, UK, 2011).

In Jordan, such a vital service does not exist as yet, highlighting the importance of conducting MMR studies on the Syrian refugees who concern an important denomination of Jordan population. Positive results anticipated from such studies would hopefully result in a similar successful service to be implemented one day for the refugees live in Jordan.

**7**. **Refugees around the World:**

Refugees has been defined by the 1951 ‘United Nations Convention on Refugees’ as people with “a well-founded fear of being persecuted for reasons of race, religion, nationality, membership of a particular social group or political opinion, is outside the country of his nationality, and is unable to, or owing to such fear, is unwilling to avail himself of the protection of that country”^[[4]](#footnote-4)^. This definition has been used in all international laws and by international standards (Bates, 2002). Refugees have to travel in order to save their lives or maintain their freedom because they have no security in their own countries; thence, if other countries do not let them in, and do not help them once they are in, then they may be judging them to death or to an unbearable life, without rights.^[[5]](#footnote-5)^

At the end of 2014, 19.5 million refugees were found worldwide with a 51% of them were under 18 years old. According to United Nations High Commissioner for Refugees (UNHCR) 2016, an average of 42,500 persons per day were forced to leave their homes and seek protection elsewhere, either within the borders of their countries or in other countries^[[6]](#footnote-6)^. Moreover, it has been estimated that 214 million people worldwide have crossed international borders for different reasons (El-Khatib *et al*., 2013). There are approximately 700 refugee camp locations all over the world (UNHCR.2015)^[[7]](#footnote-7)^. The refugee conflicts are not a contemporary occurrence all over the world, but it is new to Europe and the west where only 14% of the world’s displaced people resides in the developed world (Kingsley, 2015). Most of the world's refugees moved to developing countries 86% (UNHCR, 2016). Integration, repatriation and resettlement are the three reasonable solutions and the ultimate goal that UNHCR trying to achieve for refugees around the world.

**8. Syrian Refugees in Jordan:**

Jordan and Syria are bordering countries, with the population that speak the same language and share the same religion. Syria has a larger area and bigger total population; the total population of Jordan is 6 million and 675 thousands^[[8]](#footnote-8)^, while that of Syria is 22 million and 712 thousands^[[9]](#footnote-9)^ (World Health Organization).

Syria has become the great tragedy of this century”, says UN High Commissioner for Refugees (António Guterres). The story has begun since the crises and instability started in Syria (March 2011), where it was assessed that more than half of the 22 million Syrian population had fled their homes, of which almost 4 million people have fled seeking refuge outside Syria in neighboring countries^[[10]](#footnote-10)^. Among these countries are Lebanon, Iraq, Turkey and Jordan (Achilli, 2015). Less than 6% of the Syrian refugees had applied for asylum in Europe (Kingsley, 2015). Nowadays, Syria had become the world’s top source country of refugees with an average of almost one Syrian out of every four refugees**^[[11]](#footnote-11)^.**

Jordan has received an enormous number of Syrian refugees coming from right across the border, from the city of Deraa into northern Jordan through the towns of Jabir and Ramtha^[[12]](#footnote-12)^. With time, the situation became hypercritical as the war aggravated; an average of 1000 Syrian people began to cross the border every day^[[13]](#footnote-13)^. To overcome this high number of refugees fleeing to Jordan, many camps were established in the country^[[14]](#footnote-14)^. These camps are called Za’atari, Marjeeb al-Fahood, Cyber City and Al-Azraq camps. Za’atari refugee camp was the first that set up in July 2012 in a windswept desert. The United Nations had registered 637,859 refugees in Jordan, with over 80,000 registered in the Za’atri camp in February 2015^[[15]](#footnote-15)^. This meant that 80% of Syrian refugees in Jordan settled in urban and rural areas while the remaining 20% lived in the camps^[[16]](#footnote-16)^. The international statistics declared that 41% of the incoming refugees are children, 30% are women and 29% are men (Al-Kilani, 2014). UNHCR’s Home Visits Program Report has estimated that the largest number of Syrian refugees in Jordan are living outside these camps (84%); with approximately (33.6%) living in Amman, (28.4%) in Irbid, (13.2%) in Mafraq and (10.5%) in Zarqa^[[17]](#footnote-17)^.

Several factors are behind this large influx of Syrian refugees to Jordan such as refugees’ kinship and friendship ties with Jordan, the political stability of the Hashemite Kingdom, and the historically tight cultural and geographical relationship between Syria and Jordan (Achilli, 2015). Furthermore, Jordan has always welcomed generously different population from neighboring countries to its community, with more than 40% of Jordan’s current population arising from other countries such as Palestine (two million) and Iraq (29,000) (Al-Kilani, 2014).

**9. Health situation among the Syrian Refugees in Jordan:**

Refugees all over the world are facing several health-related problems and most of them are not gaining proper medical care, and the situation among Syrian refugees is not any different (Burgess, 2004; Pfortmueller *et al*., 2016). The load on the health system is huge in Jordan due to receiving the largest number of Syrian refugees (Arie, 2015).

In Jordan, UNHCR and other partners with the effort of the Jordanian Ministry of Health (MOH) provided health and humanitarian support for Syrian refugees that living inside and outside the camps (Murshidi *et al*., 2013). However, the larger fraction of refugees that lives outside the camps faces different health-related challenges (Ay *et al*., 2016). The needs for this population are not being sufficiently covered (Ay *et al*., 2016; El-Khatib *et al*., 2013). UNHCR-Unregistered refugees must wait for weeks to months for their interview and they need to pay out of pocket for medical services (El-Khatib *et al*., 2013). Since 2012, the Jordanian MOH provided full access to primary and secondary health centers for the Syrians living outside camps along with the local Jordanian population for all UNHCR-registered refugees (Ay *et al*., 2016; Murshidi *et al*., 2013). Once registration expires, refugees can continue to reside in Jordan but they must pay the non-residents fees in the governmental hospitals and centers (except for vaccines) (Ay *et al*., 2016; Murshidi *et al*., 2013). However, governmental health sectors provide urgent care needs only and these sectors are exhausted in many sections such as neonatal incubator, surgical operation, cancer treatment and weapons-related wounds (Murshidi *et al*., 2013; El-Khatib *et al*., 2013). Additionally, some non-governmental organizations and physicians also deliver services to Syrian refugees outside the camps (Doocy *et al*., 2016); Jordan Health AID Society is a popular example for such these organizations. The WHO declared that the number of Syrian refugees getting benefit from public hospitals in Jordan has increased lately by approximately 250% (Coutts & Fouad, 2013). As for those requiring surgical operations outside the refugee camps, such number has increased by 600% ([Coutts](javascript:void(0);) & [Fouad](javascript:void(0);), 2013).

The increasing number of Syrian refugees in Jordan and the subsequent needs for health system accessibility has resulted in a large load on the system (Doocy *et al*., 2016). The Jordanian government is in need for additional assistance to preserve the current level of health access provided to its Syrian refugees (Doocy *et al*., 2016).

The health situation for the Syrian refugees has been studied regarding many conditions such as depression, anxiety and chronic diseases (Basheti et al., 2015; Hassan *et al*., 2016; Doocy *et al*., 2016). A survey involving 1550 Syrian household refugees that conducted in 2014 in Jordan showed that 43.4% of Syrian household members have one or more of the chronic diseases such as cardiovascular disease, hypertension, arthritis, diabetes or respiratory disease^[[18]](#footnote-18)^. Moreover, it has been obtained that more than 26% of this population stopped taking their medicines for longer than two weeks due to high cost^[[19]](#footnote-19)^. More than one study has revealed that the Syrian refugees in Jordan have suffered from psychological distresses such as depression (Gammouh *et al*., 2015; Basheti *et al*., 2015).

Our humanitarian and medical role as pharmacists requires us to make an urgent intervention for the Syrian refugees that became a crucial denomination of Jordanian populations. The MMR service would be an ideal service to deliver to the Syrian refugees in Jordan for the many benefits it showed with other population. MMR in Jordan involving the Syrian refugees have to be one of the essential issues to be tackled in the pharmaceutical research at this stage. This research is going to address the impact of identifying and resolving TRPs by MMR service on many different aspects of the Syrian refugees' health outcomes. In Jordan, such a vital service does not even exist yet, highlighting the importance of conducting MMR studies in the country. Positive results anticipated from such studies would hopefully result in a similar successful service to be implemented one day.

**Methodology**:

**1. Research Hypothesis**

*Null hypothesis*: The implementation of the MMR service for adult Syrian refugees with chronic conditions in Jordan will have no impact on the number of identified TRPs, anxiety scale, self-reported adherence and quality of life in comparison to patients that will not receive this service.

*Alternative hypothesis:* The implementation of the MMR service for adult Syrian refugees with chronic conditions in Jordan will have significant impact on the number of identified TRPs, anxiety scale, self-reported adherence and quality of life in comparison to patients that will not receive this service.

**2. Ethics Approval**

Ethics approval will be obtained from The Jordanian Ministry of Health before starting the clinical work.

**3. Study Design and Clinical Setting**

The study will be conducted over 6 months from May 2016 to October 2016 in three big cities in Jordan (Amman, Mafraq, Zarqa); on Syrian refugees that reside in these two areas. The study will follow the single-blinded randomized controlled trial. Eligible patients will be randomized into two groups, intervention and control. Predetermined randomization number list which will be designed through computer-generated randomization program will be available before starting the study. The patients will be invited to participate in the study and he/she will be asked to sign the informed consent form. Only Syrian refugees who meet all of the following inclusion criteria will be recruited into the study: (1) Living in Jordan for more than six months and intending to stay for the study period which is six months. (2) Being above the age of 18 years. (3) Having at least one chronic condition or taking 5 or more medications or taking more than 12 doses of medication per day. The Exclusion criteria for this study will include: (1) Patients with cognitive or sensory impairment that may prevent conducting the interview. (2) Patients who are planning to travel within the next six months after the baseline home-visit. (3) Patients who are not capable of reading or writing Arabic.

**4. Study Protocol:** Figure (1) shows the concert for the study protocol

Patients Eligibility: patients that meet the recruitment criteria will be invited to participate in the study

The patients will be interviewed at their homes to collect all needed information. The patients will be asked to answer the self-completion questionnaires (Quality of life, scale of anxiety, knowledge of medication, adherence)

Pharmacist counseling will be conducted for intervention group only

The pharmacist will interview the patients 2-3 months post baseline

The patients in intervention group will be asked to visit the physician to confirm these changes

The approved recommendations for control group patients will be kept until the end of the study

The physician will approve part of the recommendations

A report will be written for the physician

The pharmacist will conduct the medication review for all patients

Control group

Intervention group

Randomization

Eligible patients who will fulfill the including criteria will be randomized into two groups, intervention and control groups. Patients will be informed on the nature of the study and will be asked to sign informed consent form. Patients in both intervention and control groups will be interviewed at their homes to assess their use of the treatment and to collect all relevant information. In addition, they will be asked to answer the quality of life questionnaire, the self-reported adherence questionnaire, knowledge about medication questionnaire and the scale of anxiety questionnaire. Medication review (explained in the next section [5]) will be conducted for both intervention and control groups. Only patients in intervention group will receive counseling that does not exceed the clinical pharmacist role (explained in the next section [6]). This will be followed by writing a report to the physician with the findings and recommendations. The report will be delivered to the physician directly with references supported by current guidelines. The physician will tick the recommendations approved and return to the researcher, allowing the clinical pharmacist to convey the approved changes to the patient. The patients will be asked to visit their physician for confirmation of changes. Patients in the active and the control group will be interviewed 2-3 months post baseline, assessing changes in treatment, number of treatment related problems, changes in the quality of life, changes in self-reported adherence, changes in anxiety level and changes in patients’ knowledge about their medication. Patients in the intervention group will provide their perspective of MMR service (role of the pharmacist, value of the service they received, and willingness to pay for such services).

As for the control group, home interview will also be conducted, but no intervention (patient counseling or returning the physician's report) will be done until the end of the study. Questions from control group patients will be recorded and answered at the end of the study. The recommendations that approved by the physician concerning the control group will be returned back to them directly after the study period finished. The pharmacist will ask the patients in the control group to visit the physician to confirm these changes. Patients in control group with life-threatening TRPs will be excluded from the study (serious drug-drug interactions, medication high dose that may damage kidney or liver function over time, serious drug-disease interaction, pregnancy-related problems (including mother and fetus), occurrence of sudden health episode or complication during the study period (Carolan *et al*., 2016)).

**5. Medication Review:**

The Australian model for medication review service will be followed in this study (since this service does not exist yet in the country). The clinical pharmacist will interview the patients at their own homes; collect all the information relevant to their medications and health conditions including current diseases, current drugs, history of previous diseases and drugs, side effects and allergy. This will be followed by interpretation and analyzing of the information regarding the patient’s medical history, current therapeutic regimen along with any needs and concerns; identifying therapeutic and other issues relevant to the short and long term management of the patient; making appropriate recommendations regarding the clinical information gathered for consideration by a physician.

**6. Pharmacist’s Role in Medication Review**

The pharmacist will provide proper education and counseling for patients regarding their illnesses and medications. In addition, information about the importance of adherence to the therapy will be provided to the patients. Advices regarding self-treatment and herbal remedies abuse will be explained to the patients. Effective, open-ended questioning and active listening will be used in order to achieve proper education for the patients. A patient will learn best by hearing spoken instructions and by directly handling medications.

**7. Baseline Assessments**

At baseline, patients from control and intervention groups will be interviewed at their homes to assess their use of treatment and to collect all relevant information regarding patients' demographics, current diseases, current drugs, history of previous diseases and drugs, side effects and allergy (Appendix 5). In addition, Patients' adherence, anxiety level, quality of life and medication-knowledge level will be assessed (Figure 1).

**8. Follow-up Assessments**

Both groups will be revisited at their homes 2-3 months after the baseline assessment. This assessment traces changes in treatment, number and type of TRPs, adherence, anxiety, quality of life and patients’ knowledge about medication (Figure 1). A collective report highlighting the feasibility and success of the study will be submit to the involved physicians three months following the end of the study.

**9. Adherence to Medication [Appendix 1]**

Questionnaire will be conducted for the patients to assess the patients’ self-reported adherence to their medications. AbuRuz et al. (2011) developed this questionnaire depending on a scale that was developed by Morisky et al. (1986). The questionnaire consists of five items including questions of how often during the last month the patients forgot to take their medication, skipped their medication, stopped their medication when they felt better, stopped their medication when they felt worse or stopped their medication when they experienced side effect. The measurement scale used in this questionnaire is scored at 0 (never), 1 (rarely), 2 (sometimes), 3 (often) and 4 (always). Hence, adherence will be analyzed as a continuous scale out of five (the higher the score is, the less the patient is adherent).

**10. Quality of Life [Appendix 2]**

All patients will be asked to answer the self-completion Quality of Life Questionnaire (EQ-5D questionnaire) which is one of the most commonly used questionnaires to measure health-related quality of life. This questionnaire consists of 5 domains (mobility, self-care, usual activities, pain/discomfort and anxiety/ depression) in which each domain has 5 levels of severity; no problem (level 1), mild problem (level 2), moderate problem (level 3), severe problem (level4) and extreme problem‖ (level5).

**11. Medication knowledge [Appendix 3]**

Patients' knowledge about medication will be assessed by the Knowledge about Drug Therapy Questionnaire that is validated by AbuRuz et al (2011). The questionnaire consists of four questions related to patients' medications including (How, When, Why) is he/she taking the medication, and any other comments they may have related to this medication .

**12. Scale of Anxiety [Appendix 4]**

Patient’s scale of anxiety will be assessed using a questionnaire composed of seven items relevant to generalized anxiety Zigmond & Snaith (1983). The questionnaire is scored at a scale of 4 (0-3).The possible score for each patient will range from 0 t0 21. Hence, the anxiety will be analyzed as a continuous scale out of four (the higher the score is, the more the patient is anxious).

**13. Definition and Classification of TRPs**

AbuRuz *et al*. (2006) classification system will be used in order to identify the TRPs which actually or potentially interfere with the clinical outcomes for each patient. This classification has been widely implemented and carefully explained in prior research (Basheti *et al*.,2013; Basheti *et al*., 2016). In this study, this classification will be used to identify unnecessary drug therapy, untreated conditions, ineffective/incomplete drug therapy, inappropriate dosage regimen, adverse drug effects, actual or potential drug interactions, non-adherence and suboptimal monitoring.

**14. Physicians’ Acceptance of the Clinical Pharmacist Recommendations**

Many physicians will be contacted regarding the TRPs identified for the Syrian patients. This is because different refugees visit different physicians depending on the refugees’ living areas and on the medical organizations that each refuge is visiting. At the end of the study, the total number of the physicians that accept the pharmacist recommendation will be divided by the total number of the contacted physicians to calculate the acceptance rate of the physicians for the pharmacist recommendations.

**15. physicians’ approval rate of the Pharmacist Recommendations**

After the medication review, all the pharmacist’s recommendations will be sent to the physicians to take the approval from them. The physicians will approve some of these recommendations. To calculate the approval rate for the pharmacist recommendation, the total number of approved recommendations by physicians will be divided by the total number of all recommendations (approved + rejected) sent to the physicians.

**16. Sample size**

Sample size determination will be based on the primary outcome variable of TRP improvement pre and post receiving the MMR service. Based on previously published study in this area, and in order to detect a significant different change in TRPs of 1 point difference (Molino *et al*., 2014), with a significance level of 5% and power of 80%, with the standard deviation of the change being 2.92 (variance based on the data from previously published study (Basheti *et al*., 2016)), the minimum required sample size needed will be 138 patients.

**References:**

- A guide to medication review: the agenda for patients, practitioners and managers. Task Force on Medicines Partnership and The National Collaborative medicines Management Services Programme. 2002
- Abughosh, S. M., Wang, X., Serna, O., Henges, C., Masilamani, S., James Essien, E. & Fleming, M. (2016). A Pharmacist Telephone Intervention to Identify Adherence Barriers and Improve Adherence Among Nonadherent Patients with Comorbid Hypertension and Diabetes in a Medicare Advantage Plan. *Journal of Managed Care & Specialty Pharmacy*, *22*(1), 63-73.‏
- AbuRuz, S. M., Alrashdan, Y., Jarab, A., Jaber, D. and Alawwa, I. A. (2013), Evaluation of the impact of pharmaceutical care service on hospitalized patients with chronic kidney disease in Jordan. **Int J Clin Pharm**, 35: 780-789. TRP
- ‏AbuRuz, S. M., Bulatova, N. R., & Yousef, A. M. (2006). Validation of a comprehensive classification tool for treatment-related problems. *Pharmacy World and Science*, *28*(4), 222-232.‏
- AbuRuz, S. M., Bulatova, N. R., Yousef, A. M. M., Al-Ghazawi, M. A., Alawwa, I. A., & Al-Saleh, A. (2011). Comprehensive assessment of treatment related problems in hospitalized medicine patients in Jordan. *International journal of clinical pharmacy*, *33*(3), 501-511.‏ TRP
- AbuRuz, S., Al‐Ghazawi, M., & Snyder, A. (2012). Pharmaceutical care in a community‐based practice setting in Jordan: where are we now with our attitudes and perceived barriers?. *International Journal of Pharmacy Practice*, *20*(2), 71-79.‏
- Achilli, L., 2015. Syrian Refugees in Jordan: a Reality Check. Migration Policy Centre, EUI. European University Institute. DOI 10.2870/821248
- ALBashtawy, M., Batiha, A. M., Tawalbeh, L., Tubaishat, A., & AlAzzam, M. (2015). Self-medication among school students. *The Journal of School Nursing*, 31(2), 110-116.‏
- Al-Eidan, F. A., McElnay, J. C., Scott, M. G., McConnell, J. B. (2002). Management

of Helicobacter pylori eradication – the influence of structured counselling and follow-up. Br Jornal Clinical Pharmacology, 53, pp163–71.

- Al-Kilani, S., 2014. A duty and a burden on Jordan. Forced Migration Review, Available at: <http://www.fmreview.org/syria/alkilani.html> Accessed at: 28 Feb 2016.
- American Pharmacists Association (APhA) (2004), Medication Therapy Management Services Definition and Program Criteria.
- American Pharmacists Association and National Association of Chain Drug Stores Foundation (NACDS and APhA) (2008), Medication Therapy Management in Pharmacy Practice Core elements of MTM Service Model
- American Society of Health-System Pharmacists, 2015. Available at: http://www.ashp.org
- Arie, S., 2015. Syrian doctors risk arrested and deportation for treating fellow refugees in Lebanon and Jordan. BMJ, 350, pp.1552.
- Aronson, J. K. (2009). Medication errors: definitions and classification. *British Journal of Clinical Pharmacology*, *67*(6), pp. 599–604.
- Australian Government Medicare Australia. http://www.medi careaustralia.gov.au/provider/pbs/fifth-agreement/residentialmedication-management-review.jsp. Accessed March 2016.
- Awad, A., Eltayeb, I., Matowe, L., & Thalib, L. (2005). Self-medication with antibiotics and antimalarials in the community of Khartoum State, Sudan. *J Pharm Pharm Sci*, *8* (2), 326-331.‏
- Ay, M., González, A., Delgado, C., (2016). The Perceived Barriers of Access to Health Care Among a Group of Non-camp Syrian Refugees in Jordan. International Journal of Health Services, 9, pii: 0020731416636831
- Basheti, I.A., El Hait, S.S., Qunaibi, E.A., Aburuz, S. and Bulatova, N., 2016 a. Associations between patient factors and medication adherence: A Jordanian experience. Pharmacy Practice, 14(1), pp.639. (a)
- Basheti, I.A., Obeidat, M.N., AL-Qudah, R.A., Bulatova, N.R., (2016 b). Home medication management review in outpatients with chronic diseases in Jordan: a randomized control trial. International Journal of Clinical Pharmacy, 10.1007/s11096-016-0266-9, pp.1-10.
- Basheti, I.A., Qunaibi, E.A., Bulatova, N.R., Samara, S., AbuRuz, S., (2013). Treatment related problems for outpatients with chronic diseases in Jordan: the value of home medication reviews. International Journal of Clinical Pharmacy, 35, pp.92–100
- Basheti, I.A., Qunaibi, E.A., Hamadi, S.A., Abu-Gharbieh, E., Saleh, S., AbuRuz, S., Mohamoud, M. and Bulatova, N.R., (2014). Patient Perspectives of the Role of the Community Pharmacist in the Middle East: Jordan, United Arab Emirates and Iraq. Pharmacology & Pharmacy, 5(6), p.588.
- Basheti, I.A., Qunaibi, E.A., Malas, R., (2015). Psychological Impact of Life as Refufees:A Pilot Study on a Syrian Camp in Jordan. Tropical Journal of Pharmaceutical Research, 14(9), pp.1695-1701
- Basheti, I.A., Tadros, O.K. and Aburuz, S., 2016 c. Value of a Community‐Based Medication Management Review Service in Jordan: A Prospective Randomized Controlled Study. Pharmacotherapy: The Journal of Human Pharmacology and Drug Therapy.
- Bates, D.C., 2002. Environmental refugees? Classifying human migrations caused by environmental change. Population and environment, 23(5), pp.465-477.
- Bates, D.W., Boyle, D.L., Vliet, M.B.V., Schneider, J., Leape, L., (1995). Relationship between medication errors and adverse drug events. Journal of General Internal Medicine, 10(4), PP.199-205
- Bergheim, S., Jacobsen, C.D., Clausen, F. and Straand, J., (2008). Home visits by a pharmacist after discharge from hospital. Tidsskr Nor Laegeforen, 128(5), pp.567-569
- Bjelland, I., Dahl, A.A., Haug, T.T. and Neckelmann, D., 2002. The validity of the Hospital Anxiety and Depression Scale: an updated literature review.Journal of psychosomatic research, 52(2), pp.69-77.
- Blenkinsopp, A., Bond, C., Raynor, D.K., (2012). Medication reviews. British Journal of Clinical Pharmacology, 74(4), pp.573–80
- Bruhn, H., Bond, C.M., Elliott, A.M., Hannaford, P.C., Lee, A.J., McNamee, P., Smith, B.H., Watson, M.C., Holland, R. and Wright, D., 2013. Pharmacist-led management of chronic pain in primary care: results from a randomized controlled exploratory trial. BMJ open, 3(4), p.p 2361.
- Brulhart, M.I., Wermeille, J.P., 2011. Multidisciplinary medication review: evaluation of a pharmaceutical care model for nursing homes. International Journal of Clinical Pharmacy, Vol 33(3), pp. 549-557 Type of Interventions and num. of TRP
- Brummel, A., & Carlson, A. M., (2016). Comprehensive Medication Management and Medication Adherence for Chronic Conditions. Journal of Managed Care & Specialty Pharmacy, 22(1), pp.56-62.‏ Adherence improvement
- Burgess, A., (2004). Health Challenges for Refugees and Immigrants. Refugees report, 25(2)
- Cassedy, P., (2010). First steps in clinical supervision: A guide for healthcare professionals. Maidenhead, Berkshire. McGraw-Hill Open University Press.
- Castelino, R.L., Bajorek, B.V., Chen, T.F., (2009). Targeting suboptimal prescribing

in the elderly: a review of the impact of pharmacy services. Annual Pharmacotherapy, 43(6), pp.1096–106.

- Castelino, R. L., Bajorek, B. V., Chen, T. F., (2011). Are interventions recommended by pharmacists during Home Medicines Review evidence‐based?. Journal of evaluation in clinical practice, 17(1), pp.104-110.
- Cheragi, M.A., Manoocheri, H., Mohammadnejad, E., Ehsani, S.R., (2013). Types and causes of medication errors from nurse's view-point. Iranian Journal of Nursing Midwifery, 18(3), pp. 228–231.
- Chisholm-Burns, M. A., Lee, J. K., Spivey, C. A., Slack, M., Herrier, R. N., Hall-Lipsy, E., Kramer, S. S., (2010). US pharmacists' effect as team members on patient care: systematic review and meta-analyses. Medical care, 48(10), pp. 923-933.‏
- Chow, E.P., Hassali, M.A., Saleem, F. and Aljadhey, H., 2015. Effects of pharmacist-led patient education on diabetes-related knowledge and medication adherence: A home-based study. Health Education Journal, p.0017896915597021.
- Cobb, M.D., (1986). Evaluating medication errors. Journal of Nursing Administration, 16(4), pp.41-44.‏
- Cohen, M. (2007). Medication errors. 2nd ed. Washington, DC, American Pharmacists Association.
- Coutts, A., & Fouad, F. M., (2013). Response to Syria's health crisis—poor and uncoordinated. The Lancet, 381(9885), pp. 2242-2243.
- de Lyra, D.P., Kheir, N., Abriata, J.P., da Rocha, C.E., dos Santos, C.B. and Pelá, I.R., 2007. Impact of pharmaceutical care interventions in the identification and resolution of drug-related problems and on quality of life in a group of elderly outpatients in Ribeirão Preto (SP), Brazil. Therapeutics and clinical risk management, 3(6), p.989. Harvard style
- Dolan, P., Gudex, C., Kind, P. and Williams, A., (1995). A social Traiff for EuroQol: Results from a UK general population survey. The University of York.
- Doocy, S., Lyles, E., Akhu‐Zaheya, L., Burton, A., & Weiss, W., (2016). Health service utilization and access to medicines among Syrian refugee children in Jordan. The International Journal of Health Planning and Management, 31(1), pp.97-112.
- Doocy, S., Lyles, E., Roberton, T., Akhu-Zaheya, L., Oweis, A. and Burnham, G., 2015. Prevalence and care-seeking for chronic diseases among Syrian refugees in Jordan. BMC public health, 15(1), p.1097 harvard style
- Dood, C., (2006). Assessment pharmacy intervention at salisbury. Health Care NHL Trust. American Journal of Hospital Pharmacy, 10, pp. 451-456.
- El-Khatib, Z., Scales, D., Vearey, J., Forsberg, B. C., (2013). Syrian refugees, between rocky crisis in Syria and hard inaccessibility to healthcare services in Lebanon and Jordan. Conflict and health, 7(1), pp. 1.
- Elliott, R.A., Lee, C.Y., Beanland, C., Vakil, K., Goeman, D., (2016). Medicines Management, Medication Errors and Adverse Medication Events in Older People Referred to a Community Nursing Service: A Retrospective Observational Study. Drugs-Real World Outcomes, 3(1), pp.13-24.‏
- Ernst, F. R., & Grizzle, A. J. (2001). Drug-related morbidity and mortality: updating the cost-of-illness model. *JAPHA-WASHINGTON-*, *41*(2), 192-199.‏
- Facts and Figures about Refugees. UNHCR, 2016. [Accessed at March 7th 2016]. Available at: <http://www.unhcr.org.uk/about-us/key-facts-and-figures.html>.
- Fakeye, T. O., Adisa, R., & Musa, I. E. (2009). Attitude and use of herbal medicines among pregnant women in Nigeria. *BMC Complementary and Alternative Medicine*, *9* (1), 53.‏
- Ferner, R.E. and Aronson, J.K., (2006). Clarification of terminology in medication errors. *Drug safety*, *29*(11), pp.1011-1022.
- Ferner, R.E., Aronson, J.K., (2006). Clarification of terminology in medication errors: definitions and classification. Drug Safety, 29 (11), pp.1011-1022.
- Freeman, C.R., Cottrell, W.N., Kyle, G., Williams, I.D. and Nissen, L., 2013. An evaluation of medication review reports across different settings. International journal of clinical pharmacy, 35(1), pp.5-13.
- Gammouh, O. S., Al-Smadi, A. M., Tawalbeh, L. I., & Khoury, L. S., (2015). Peer Reviewed: Chronic Diseases, Lack of Medications, and Depression Among Syrian Refugees in Jordan, 2013–2014. Preventing chronic disease, 12
- Gilbert, A.L., Roughead, E.E., Beilby, J., Mott, K. and Barratt, J.D., (2002). Collaborative medication management services: improving patient care. Medical Journal of Australia, 177(4), pp. 189-192.
- Hassan, G., Ventevogel, P., Jefee-Bahloul, H., Barkil-Oteo, A., Kirmayer, L. J., (2016). Mental health and psychosocial wellbeing of Syrians affected by armed conflict. Epidemiology and psychiatric sciences, 25(02), pp.129-141.‏
- Haugbolle, L., S., Sorensen, E., W., (2006). Drug-related problems in patients with angina pectoris, type 2 diabetes and asthma--interviewing patients at home. Pharmacy World and Science, 28, pp.239-247.
- Hepler, C. D., & Strand, L. M. (1990). Opportunities and responsibilities in pharmaceutical care. *Am J hosp pharm*, *47*(3), 533-543.‏
- Holland, R., Lenaghan, E., Harvey, I., Smith, R., Shepstone, L., Lipp, A., Christou, M., Evans, D. and Hand, C. (2005). Does home based medication review keep older people out of hospital? The HOMER randomised controlled trial. **Bmj**, 330, pp293.
- Institute of medicine [IOM].1999. Available at: iom.nationalacademies.org
- Jassim, A.M., (2010). In-home Drug Storage and Self-medication with Antimicrobial Drugs in Basrah, Iraq. Oman Medical Journal, 25(2), pp. 79–87.
- Khan, A.R., Al-Abdul Lateef, Z.N., Al Aithan, M.A., Bu-Khamseen, M.A., Al Ibrahim, I., Khan, S.A., (2012). Factors contributing to non-compliance among diabetics attending primary health centers in the Al Hasa district of Saudi Arabia. Journal of family and community medicine, 19(1), pp26-32
- Kingsley, P., (2015). What caused the refugee crisis?. The Guardian, Available at: <http://www.theguardian.uk-news>.
- Kolhatkar, A., Cheng, L.,Chan, F., Harrison, M., Law, M., (2016). The impact of medication reviews by community pharmacists. Journal of the American Pharmacists Association 56(5), pp513-520.
- Kothari, D., Gupta, S., Sharma, C., Kothari, S., (2010). Medication error in anaesthesia and critical care. A cause for concern. Indian Journal of Anaesthesia, 54(3), pp.187–192.
- Leape, L.L., (1994). Error in medicine. JAMA, 272(23), pp.1851-1857
- Mahdikhani, S., Dabaghzadeh, F., (2016). Benefits of Pharmacist’s Participation on Hospitalist Team. Acta Medica Iranica, 54(2), pp.140-145.‏
- ‏McGivney, M. S., Meyer, S. M., Duncan-Hewitt, W., Hall, D. L., Goode, J., & Smith, R. B., (2007). Medication therapy management: its relationship to patient counseling, disease management, and pharmaceutical care. JAPHA-WASHINGTON, 47(5), pp. 620.
- Molino, Cde.G., Cavalcanti, R., Rodrigues, A.T., Visacri, M.B., Moriel, P., Mazzola, P.G. (2014). Impact of pharmacist interventions on drug-related problems and laboratory markers in outpatients with human immunodeficiency virus infection. Therapeutics and clinical risk management, 10, pp.631-639.
- Morimoto, T., Gandhi, T.K., Seger, A.C., Hsieh, T.C. and Bates, D.W., (2004). Adverse drug events and medication errors: detection and classification methods. *Quality and safety in health care*, *13*(4), pp.306-314.
- Morisky, D. E., Green, L.W., Levine, D.M. (1986). Concurrent and predictive validity of a self-reported measure of medication adherence. Medical care, 24(1), pp.67-74.‏
- Murshidi, M.M., Hijjawi, M.Q.B., Jeriesat, S., Eltom, A., (2013). Syrian refugees and Jordan’s health sector. Lancet, 382, pp.206-207. 10.1016/S0140-6736(13)61506-8.
- National Heart, Lung, and Blood Institute (2015), Classification of Overweight and Obesity by BMI, Waist Circumference, and Associated Disease Risks.
- Nebeker, J.R., Barach, P., Samore, M.H., (2004). Clarifying adverse drug events: a clinician's guide to terminology, documentation, and reporting. Annals of internal medicine, 140(10), pp.795-801.‏
- NHS Cumbria Medicines Management Team (2011). A practice guide. Available at: [http://www.cumbria.nhs.uk/ProfessionalZone/Medicines Management/Guidelines/MedicationReview-PracticeGuide2011.pdf](http://www.cumbria.nhs.uk/ProfessionalZone/Medicines%20Management/Guidelines/MedicationReview-PracticeGuide2011.pdf)
- O’Shea, E., (1999). Factors contributing to medication errors: a literature review. *Journal of clinical nursing*, *8*(5), pp.496-504.
- Obradovic, M., Lal, A. and Liedgens, H., 2013. Validity and responsiveness of EuroQol-5 dimension (EQ-5D) versus Short Form-6 dimension (SF-6D) questionnaire in chronic pain. Health and quality of life outcomes, 11(1), p.1.
- Oladapo, A. O., & Rascati, K. L., (2012). Review of survey articles regarding medication therapy management (MTM) services/programs in the United States. Journal of pharmacy practice, 0897190012442715.‏
- Oliveira, D.R., Brummel, A.R., Miller, D.B., (2010). Medication Therapy Management: 10 Years of Experience in a Large Integrated Health Care System. Journal of managed care pharmacy, 16(3), pp.185-95
- Olson, M.D., Tong, G.L., Steiner, B.D., Viera, A.J., Ashkin, E., Newton, W.P., (2012). Medication documentation in a primary care network serving North Carolina medicaid patients: results of a cross-sectional chart review. BMC Family Practice, 13, pp.1
- Otoom, S.A. and Sequeira, R.P., (2006). Health care providers' perceptions of the problems and causes of irrational use of drugs in two Middle East countries. *International journal of clinical practice*, *60*(5), pp.565-570.
- Patel, P., & Zed, P. J. (2002). Drug‐Related Visits to the Emergency Department: How Big Is the Problem?. *Pharmacotherapy: The Journal of Human Pharmacology and Drug Therapy*, *22*(7), 915-923.‏
- Pellegrino, A. N., Martin, M. T., Tilton, J. J., & Touchette, D. R. (2009). Medication therapy management services. *Drugs*, *69*(4), 393-406.‏
- Pfortmueller, C.A., Schwetlick, M., Mueller, T., Lehmann, B., Exadaktylos, A.K., (2016). Adult Asylum Seekers from the Middle East Including Syria in Central Europe: What Are Their Health Care Problems?. PLoS ONE, 11(2), doi:10.1371/journal.pone.0148196
- Pharmaceutical Care Network Europe (PCNE). [Accessed at March 13, 2016]. Available at: http://www.pcne.org/working-groups/2/drug-related-problems.
- Phillips, D.P., Bredder, C.C., (2002). Morbidity and mortality from medical errors. An increasingly serious public health problem. Annual RevIEW of Public Health, 23:135–150
- Piette, J. D., Heisler, M., Krein, S., Kerr, E. A., (2005). The role of patient-physician trust in moderating medication non-adherence due to cost pressures. Archives of internal medicine, 165(15), pp.1749-1755.
- Pinilla, J., Murillo, C., Carrasco, G. and Humet, C., (2006). Case-control analysis of the financial cost of medication errors in hospitalized patients. *The European Journal of Health Economics*, *7*(1), pp.66-71.
- Rao, D., Gilbert, A., Strand, L. M. and Cipolle, R. J. (2007), Drug therapy problems found in ambulatory patient populations in Minnesota and South Australia. Pharmaceutical World Science, 29(6): 647-654
- Refugee crisis was caused by a careless West that allowed anarchy and fear to take root in the Middle East, 2015. Independent. [Accessed at: 7 March 2016]. Available at: <http://www.independent.co.uk/news/world/middle-east/refugee-crisis-was-caused-by-a-careless-west-that-allowed-anarchy-and-fear-to-take-root-in-the-10509173.html>
- Riley, K. (2013), Enhanced medication management in the community: a win-win proposal from an economic, clinical and humanistic perspective. Can Pharm J (Ott), 146-162
- Río-Lanza, A.B., Suárez-Álvarez, L., Suárez-Vázquez, A., Vázquez-Casielles, R., (2016). Information provision and attentive listening as determinants of patient perceptions of shared decision-making around chronic illnesses. SpringerPlus, 5(1), pp.1386.
- Roughead, E. E. (1999). The nature and extent of drug‐related hospitalisations in Australia. Journal of quality in clinical practice, 19(1), 19-22.‏
- Roughead E.E., Barratt, J.D., Gilbert, A.L., 2004. Medication-related problems

commonly occurring in an Australian community setting. Pharmacoepidemiology

Drug Safety. 13(2), pp.83–7.

- Scottish Pharmacy in Mental Health, 2005. Available at :

http://www.nes.scot.nhs.uk/media/415392/nes_mental_pharmacy_-_final.pdf

- Sculpher, M., 2006. The use of quality-adjusted life-years in cost-effectiveness studies. Journal of Allergy, 61(5), pp.527-530.
- Stafford, A. C., Tenni, P. C., Peterson, G. M., Jackson, S. L., Hejlesen, A., Villesen, C., & Rasmussen, M., (2009). Drug-related problems identified in medication reviews by Australian pharmacists. Pharmacy world & science, 31(2), pp.216-223.
- Stausberg, J., & Hasford, J. (2011). Drug-related admissions and hospital-acquired adverse drug events in Germany: a longitudinal analysis from 2003 to 2007 of ICD-10-coded routine data. *BMC health services research*, *11*(1),1.‏
- Syrian Refugee Health Access Survey in Jordan December (2014). UNHCR, John Hopkins, Jordan University of Science & Technology, World Health Organization. [Accessed at 29 Feb 2016]. Available at: <file:///C:/Users/User/Downloads/JordanHealthAccessSurveyReport(FINAL).pdf>
- Syrian Refugees, A snapshot of the crisis – in the Middle East and Europe, 2016. [Accessed at: 17^th^ March 2016]. Available at: <http://syrianrefugees.eu/?page_id=87>
- Tan, X.I., Patel, I. and Chang, J., 2014. Review of the four item Morisky medication Adherence Scale (MMAS-4) and eight item Morisky medication Adherence Scale (MMAS-8). INNOVATIONS in pharmacy, 5(3), p.p 5.
- The American Pharmacists Association and the National Association of Chain Drug Stores Foundation, 2010.
- The Royal Pharmaceutical Society of Great Britain (RPSGB), department of health, (2001).
- UNHCR, 2015. Syria Regional Refugee Response: Inter-agency .Information Sharing Portal, http://data.unhcr.org/syrianrefugees/country , accessed at 28 Feb 2016.
- Viktil, K. K., & Blix, H. S. (2008). The impact of clinical pharmacists on drug‐related problems and clinical outcomes. *Basic & clinical pharmacology & toxicology*, *102*(3), 275-280.‏
- Voon, F., Keynes, S., 2014. Living in the Shadow. UNHCR. Jordan Home Visit Reports. Accessed at 28/2/2016. Available at: <http://www.unhcr.org/54b685079.pdf> .
- Wazaify, M., Al-Bsoul-Younes, A., Abu-Gharbieh, E., Tahaineh, L., (2008). Societal perspectives on the role of community pharmacists and over-the-counter drugs in Jordan. Pharmacy World and Science, 30, pp.884-889
- Westerlund, T., & Marklund, B., (2009). Assessment of the clinical and economic outcomes of pharmacy interventions in drug‐related problems. *Journal of clinical pharmacy and therapeutics*, *34*(3), 319-327.‏
- Wilkinson, D. (2005). The essential guide to postgraduate study. Sage.‏
- Woodward, H. I., Mytton, O. T., Lemer, C., Yardley, I. E., Ellis, B. M., Rutter, P. D., Wu, A. W., (2010). What have we learned about interventions to reduce medical errors?. Annual review of public health, 31, pp.479-497.‏
- World Health Organization, 2016. [Accessed at 29 Feb 2016]. Available at: <http://www.emro.who.int/jor/who-presence-in-jordan/>
- World Health Organization, 2016. [Accessed at 29 Feb 2016]. Available at: <http://www.emro.who.int/countries/syr/index.html>
- Yin, R. K., (2013). Case study research: Design and methods. Sage publications.
- Yusuff, K.B., Tayo, F., (2011). Frequency, types and severity of medication use-related problems among medical outpatients in Nigeria. International Journal of Clinical Pharmacy, 33(3), pp.558-564
- Zigmond, A. S., & Snaith, R. P., (1983). The hospital anxiety and depression scale. Acta psychiatrica scandinavica, 67(6), pp.361-370.

**(Appendix1) Medication Adherance Questionnaire**

**الناس عادة يواجهون عدة مشاكل أثناء تناولهم لأدويتهم الموصوفة لتعددها واختلاف اوقاتها وأشكالها. نود أن نسألك بعض الأسئلة عن طبيعة استخدامك لدوائك.**

| **دائما** | **عادة** | **أحيانا** | **نادرا** | **إطلاقا** | **الأسئلة** |  |
| --- | --- | --- | --- | --- | --- | --- |
|  |  |  |  |  | **هل تنسى تناول دوائك؟** | **1** |
|  |  |  |  |  | **هل تتوقف عن تناول دوائك من وقت لآخر؟** | **2** |
|  |  |  |  |  | **هل تتوقف عن تناول دوائك عندما تشعر بتحسن؟** | **3** |
|  |  |  |  |  | **هل تتوقف عن تناول دوائك إذا ساءت حالتك بعد أخذ الدواء؟** | **4** |
|  |  |  |  |  | **هل تتوقف عن تناول دوائك إذا حصلت لك مضاعفات تعتقد انها ناتجة عن استخدام الدواء؟** | **5** |
|  |  |  |  |  | **هل تلتزم بنصائح الطبيب الصيدلاني فيما يتعلق بغذائك,ممارسة الرياضة,التدخين,الخ..؟** | **6** |
|  |  |  |  |  | **كم مرة أسبوعيا لا تتناول دوائك؟** | **7** |
|  |  |  |  |  | **ما هي اكثر الأسباب التي تعيقك عن أخذ دوائك:(أجب بنعم أو لا)**  **-السعر**  **-الوقت**  **-النسيان**  **-لا أحب الأدوية**  **-الدواء لا يعمل**  **-المضاعفات الناتجة عن الدواء**  **-كثرة عدد الأدوية** | **8** |

**احيانا: جرعة واحدة في الأسبوع**

**عادة: جرعتين في الأسبوع**

**دائما: أكثر من جرعتين في الأسبوع**

**)Appendix2) EQ-5D Health Questionnaire**

**ضع علامة بجانب جملة بكل مجموعة في الأسفل,لتشير إلى أفضل عبارة تصف حالتك الصحية اليوم**

**القدرة على التنقل**

ليس لدي أي مشاكل عند المشي

أعاني من مشاكل طفيفة عند المشي

أعاني من مشاكل متوسطة عند المشي

أعاني من مشاكل حادة عند المشي

ليس لدي القدرة على المشي

**العناية الشخصية**

ليس لدي أي مشاكل عند الإستحمام أو ارتداء ملابسي بنفسي

أعاني من مشاكل طفيفة عند الإستحمام أو ارتداء ملابسي بنفسي

أعاني من مشاكل متوسطة عند الإستحمام أو ارتداء ملابسي بنفسي

أعاني من مشاكل حادة عند الاستحمام أو ارتداء ملابسي بنفسي

ليس لدي القدرة على الإستحمام أو ارتداء ملابسي بنفسي

**الأنشطة المعتادة (مثل العمل,لدراسة,الأعمال المنزلية,النشاطات الأسرية او الترفيهية)**

ليس لدي أي مشاكل في ممارسة نشاطاتي المعتادة

أعاني من مشاكل طفيفة في ممارسة نشاطاتي المعتادة

أعاني من مشاكل متوسطة في ممارسة نشاطاتي المعتادة

أعاني من مشاكل حادة في ممارسة نشاطاتي المعتادة

ليس لدي القدرة على ممارسة نشاطاتي المعتادة

**الألم \عدم الراحة الجسمية**

ليس لدي أي ألم أو شعور بعدم الراحة الجسمية

أعاني من ألم طفيف أو انزعاج طفيف

أعاني ن ألم متوسط أو انزعاج متوسط

أعاني من ألم حاد أو انزعاج حاد

أعاني من ألم شديد جدا أو انزعاج شديد جدا

**القلق\الإكتئاب**

لا أعاني من أي قلق أو اكتئاب

أعاني من قلق طفيف أو اكتئاب طفيف

أعاني من قلق متوسط أو اكتئاب متوسط

أعاني من قلق حاد أو اكتئاب حاد

أعاني من قلق شديد جدا أو اكتئاب شديد جدا

- نود أن نعرف مدى سوء حالتك الصحية أو سلامتها اليوم. هذاالمقياس مدرج من الرقم 0 حتى 100

- اللاقم 100 يعني **أفضل** حالة صحية يمكنك تصورها. واللرقم 0 يعني **أسوأ** حالة صحية يمكنك تصورها.

- ضع علامة (×) على المقياس للإشارة ألى حالتك الصحية اليوم.

- الآن,قم رجاء بكتابة الرقم الذي أشرت إليه على المقياس في المربع ‎

حالتك الصحية اليوم=


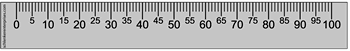
 افضل أسوأ حالة

حالة صحية

صحية

**(Appendix 3) knowledge about Drug Therapy Questionnaire**

| **ملاحظات أخرى تتعلق بالدواء** | **لماذا تأخذ الدواء** | **متى تأخذ الدواء** | **كيف تأخذ الدواء** | **الإسم العلمي** | **الإسم التجاري** |
| --- | --- | --- | --- | --- | --- |
|  |  |  |  |  |  |
|  |  |  |  |  |  |
|  |  |  |  |  |  |
|  |  |  |  |  |  |
|  |  |  |  |  |  |
|  |  |  |  |  |  |
|  |  |  |  |  |  |

**(Appendix 4) Anxiety Scale Questionnaire**

أرجو الاشارة الى الجملة التي تصف شعورك خلال الأسبوع الماضي:

1. أني أشعر بالتوتر

.3معظم الوقت .2الكثير من الوقت .1أحيانا .0إطلاقا

1. أني مازلت أستمتع بالأمور التي كانت تسعدني في الماضي

.0تماما كما في السابق .1أقل مما كنت عليه في الماضي .2قليلا .3نادرا جدا

1. يراودني شعور بالخوف وكأن هناك شيء سيء أو فظيع سوف يحصل

.3كثيرا .2عادةً .1أحيانا .0إطلاقا

1. **إني أستمتع وأضحك وأرى الجانب السعيد من كل شيء**

.0دائما قدر المستطاع .1نعم ولكن ليس كما كنت في السابق

.2القليل من الوقت .3اطلاقا

1. **هناك أفكار مريبة أو مقلقة تجول في خاطري**

.3معظم الوقت .2الكثير من الوقت .1ليس كثيرا .0قليلا جدا

1. **إني أشعر بالسعادة أو الابتهاج**

.3اطلاقاً .2نادراً .1أحياناً .0كثيراً

1. **أني أستطيع الجلوس بسلام والاسترخاء**

.0دائماً .1عادةً .2نادراً .3اطلاقا

**(Appendix 5) Patient database**

| **Name** | **Patient ID** |
| --- | --- |
| **Address** | **Name of Clinic** |
| **Phone number** | **Occupation** |
| **Date of Birth(Age)** | **Weight(Kg)** |
| **Gender** | **Educational Level** |
| **Height(cm)** | **Insurance** |
| **BMI** | **Date of the interview** |
| **IBW** | **Time of the Interview** |
| **Marital Status** | **Interview number: 1^st^ , 2^nd^** |

**History of present illness (Location, Characteristics, Aggravating and alleviating factors, Timing, Severity)**

**Past medical History/Surgery**

**Family and Social History (Diseases in first relatives,Ceregivers, Living arrangement, Daily activities)**

**Life Style (Diet, Exercise, Smoking, Alcohol, Caffeine)**

**Acute and Chronic Medical Problems (Diseases, Stage, Type, Current status, Duration)**

**(Appendix 6) treatment Related Problems (TRPs) Classification System**

| **Type of Problem** | **Assessment** | **Drug Involved/ Comment** |
| --- | --- | --- |
| **1.Unnecessary Drug therapy** | **a)Drug use without an indication**  **b) Addiction or recreational drug use**  **c) The patient treatment should be stepped down)**  **d) Duplication (two drugs from the same pharmacological class with no clinical evidence approving such combination)**  **e) Treating avoidable adverse reaction** |  |
| **2.Untreated condition** | **Untreated condition that require pharmacological or non-pharmacological therapy** |  |
| **3.Efficacy** | **a) More effective drug is available/ recommended**  **b) The patient requires additional/ combination therapy or stepping up because of actual or potential therapy failure or because of guidelines recommendation**  **c) Efficacy dosage regimen issues**  **d) Efficacy interactions issues** |  |
| **4.Safety** | **a)A current drug is contraindicated/unsafe for patient condition and should be stopped, monitored or replaced**  **b) a safer drug is recommended**  **c) The patient is at high risk for developing ADR and needs monitoring or prophylaxis**  **d) Allergic reaction or an undesirable effect: Are there symptoms or medical problems that may be drug induced?**  **e) Safety dosage regimen issues**  **f) Safety interactions issues** |  |

| **5. Inappropriate Knowledge** | **a) The patient is not instructed or does not understand important information regarding his medications (the purpose of his or her medication(s), how much, how and when to take it, what to avoid, how to prevent side effect and how to monitor his treatment)**  **b) The patient is not instructed or does not understand non-pharmacological therapy or self-care advice (avoidance of risk factors, smoking, alcohol, diet, exercise, etc.)** |  |
| --- | --- | --- |
| **6. Inappropriate adherence** | **a) A problem in patients' adherence to medications (forget, skip, cannot afford, Cannot swallow/administer drug etc)**  **b) Drug product not available**  **c) A problem in patients' adherence to self-care activities or non-pharmacological therapy** |  |
| **7.Miscellaneous** | **a) A need for additional or more frequent monitoring**  **b) A need for additional diagnostic test**  **c) A need for consultation**  **d) The chosen medication(s) is not (are not) cost effective**  **e) Other dosage regimen issues**  **f) Other interaction issues**  **g) Patient was discharged too early (i.e. before achieving recommended target)**  **h) Administering errors**  **i) Dispensing errors** |  |
| **8.Treatment Related Problem on Discharge** | **a) Unnecessary drug therapy**  **b) Untreated condition**  **c) Ineffective/incomplete drug therapy**  **d) Actual and potential ADR**  **f) Actual and potential drug interactions**  **g) Inappropriate knowledge**  **h) Inappropriate adherence**  **i) Miscellaneous** |  |

1. UNHCR, 2014. Living in the Shadow, [accessed on 28 Feb 2016]. Available at: http://www.unhcr.org/54b685079.pdf [↑](#footnote-ref-1)
2. Syrian Refugee Health Access Survey in Jordan December 2014. [Accessed at 29 Feb 2016]. Available at: <file:///C:/Users/User/Downloads/JordanHealthAccessSurveyReport(FINAL).pdf> [↑](#footnote-ref-2)
3. http://www.pcne.org/working-groups/2/drug-related-problems accessed at March 13, 2016 [↑](#footnote-ref-3)
4. http://www.who.int/topics/refugees/en [↑](#footnote-ref-4)
5. [accessed at 5 March 2016]. available at: http://www.unhcr.org/pages/49c3646c125.html [↑](#footnote-ref-5)
6. Available at: www.unhcr.org [↑](#footnote-ref-6)
7. The UN Refugee Agency" .UNHCR. Retrieved at 2015-12-18 [↑](#footnote-ref-7)
8. <http://www.emro.who.int/jor/who-presence-in-jordan/> [↑](#footnote-ref-8)
9. <http://www.emro.who.int/countries/syr/index.html> [↑](#footnote-ref-9)
10. **[Accessed at: 7 March 2016] . Available at:** [**http://www.independent.co.uk/news/world/middle-east/refugee-crisis-was-caused-by-a-careless-west-that-allowed-anarchy-and-fear-to-take-root-in-the-10509173.html**](http://www.independent.co.uk/news/world/middle-east/refugee-crisis-was-caused-by-a-careless-west-that-allowed-anarchy-and-fear-to-take-root-in-the-10509173.html) [↑](#footnote-ref-10)
11. Accessed at March 7^th^ 2016. Available at: <http://www.unhcr.org.uk/about-us/key-facts-and-figures.html> [↑](#footnote-ref-11)
12. <http://syrianrefugees.eu/?page_id=87> [↑](#footnote-ref-12)
13. <http://syrianrefugees.eu/?page_id=87> [↑](#footnote-ref-13)
14. <http://syrianrefugees.eu/?page_id=87> [↑](#footnote-ref-14)
15. UNHCR, Syria Regional Refugee Response: Inter-agency .Information Sharing Portal, <http://data.unhcr.org/syrianrefugees/country> , accessed at 28 Feb 2016. [↑](#footnote-ref-15)
16. http://syrianrefugees.eu/?page_id=87 [↑](#footnote-ref-16)
17. UNHCR, 2014. Living in the Shadow, [accessed on 28 Feb 2016].

    Available at: http://www.unhcr.org/54b685079.pdf [↑](#footnote-ref-17)
18. Syrian Refugee Health Access Survey in Jordan December 2014. [Accessed at 29 Feb 2016]. Available at: file:///C:/Users/User/Downloads/JordanHealthAccessSurveyReport(FINAL).pdf [↑](#footnote-ref-18)
19. Syrian Refugee Health Access Survey in Jordan December 2014. [Accessed at 29 Feb 2016]. Available at: <file:///C:/Users/User/Downloads/JordanHealthAccessSurveyReport(FINAL).pdf> [↑](#footnote-ref-19)
