# Supplementary material for: Improving Syrian refugees’ knowledge of medications and adherence following a randomized control trial assessing the effect of a medication management review service
Source: PLoS One. 2022 Oct 14;17(10):e0276304. doi: 10.1371/journal.pone.0276304 (PMC9565448; doi:10.1371/journal.pone.0276304)
Supplement: Attachment — Submitted filename: PONE-D-20-26688_reviewer.pdf [file pone.0276304.s004.pdf]

PLOS ONE

Improving Syrian refugees’ knowledge of medications and adherence following a randomized control trial assessing the effect of a medication management review service

--Manuscript Draft--

Manuscript Number:	PONE-D-20-26688
Article Type:	Research Article
Full Title:	Improving Syrian refugees’ knowledge of medications and adherence following a randomized control trial assessing the effect of a medication management review service
Short Title:	Pharmacists improving Refugees’ adherence and knowledge of their chronic medications
Corresponding Author:	Iman Basheti<br>Applied Science Private University Faculty of Pharmacy<br>Amman, Shafa Badran JORDAN
Keywords:	Refugees; Medication management review; adherence; chronic medication; Pharmaceutical care; Jordan; SDG 3; universal health coverage.
Abstract:	Background: Syrian refugees living in Jordan have many chronic conditions and use many medications. Pharmacists delivering the Medication Management Review (MMR) service can have a role in improving this growing global refugees’ problem.<br>Objectives: To assess the effect of the MMR service on adherence to treatment therapy and knowledge about chronic medications for Syrian refugees residing in Jordan.<br>Methods: This randomized intervention control single-blinded study was conducted in Jordan. Syrian refugees were recruited and randomized into intervention and control groups. Two home visits were delivered to each participant, at baseline and three months later. All participants completed questionnaires regarding adherence and knowledge. As a part of the MMR service, treatment-related problems (TRPs) were recognized for all patients; recommendations to resolve these TRPs were only delivered to intervention group refugees’ physicians; TRPs were corrected. At follow-up, TRPs assessment, adherence and mediation knowledge were assessed for all refugee participants.<br>Results: Participants (n=106; intervention n=53, control n=53) had a number of medications and diagnosed chronic diseases of 5.8 ± 2.1 and 2.97 ± 1.16 per participant respectively. At follow-up, a significant decrease in the number of TRPs for refugees in the intervention group was found ((from 600 to 182, P <0.001), but not for control group (number stayed at 541 TRPs, P= 0.116). A significant improvement in the adherence and knowledge scores were noted in the intervention (P < 0.001 for both) but not the control group (P= 0.229, P= 0.07 respectively).<br>Conclusion: The MMR service can significantly improve refugees’ TRPs, adherence to therapy and knowledge about chronic medications. If this approach was extended to the large scale, many refugees in need would be able to access a quality essential health-care service; a step towards achieving universal health coverage.
Order of Authors:	Majdoleen Al alawneh.
	Alberto Berardi.
	Nabeel Nuaimi
	Iman Basheti
Additional Information:	
Question	Response
Financial Disclosure<br><br>Enter a financial disclosure statement that	The author(s) received no specific funding for this work.

Powered by Editorial Manager® and ProduXion Manager® from Aries Systems Corporation

describes the sources of funding for the work included in this submission. Review the submission guidelines for detailed requirements. View published research articles from *PLOS ONE* for specific examples.<br><br>This statement is required for submission and **will appear in the published article** if the submission is accepted. Please make sure it is accurate.<br><br>**Unfunded studies**<br>Enter: *The author(s) received no specific funding for this work.*<br><br>**Funded studies**<br>Enter a statement with the following details:<br>• Initials of the authors who received each award<br>• Grant numbers awarded to each author<br>• The full name of each funder<br>• URL of each funder website<br>• Did the sponsors or funders play any role in the study design, data collection and analysis, decision to publish, or preparation of the manuscript?<br>• **NO** - Include this sentence at the end of your statement: *The funders had no role in study design, data collection and analysis, decision to publish, or preparation of the manuscript.*<br>• **YES** - Specify the role(s) played.<br><br>* typeset	
**Competing Interests**<br><br>Use the instructions below to enter a competing interest statement for this submission. On behalf of all authors, disclose any competing interests that could be perceived to bias this work—acknowledging all financial support and any other relevant financial or non-financial competing interests.<br><br>This statement **will appear in the published article** if the submission is accepted. Please make sure it is accurate. View published research articles from *PLOS ONE* for specific examples.	The authors have declared that no competing interests exist.

Powered by Editorial Manager® and ProduXion Manager® from Aries Systems Corporation

**NO authors have competing interests**<br><br>Enter: *The authors have declared that no competing interests exist.*<br><br>**Authors with competing interests**<br><br>Enter competing interest details beginning with this statement:<br><br>*I have read the journal's policy and the authors of this manuscript have the following competing interests: [insert competing interests here]*<br><br>* typeset	
**Ethics Statement**<br><br>Enter an ethics statement for this submission. This statement is required if the study involved:<br><br>• Human participants<br>• Human specimens or tissue<br>• Vertebrate animals or cephalopods<br>• Vertebrate embryos or tissues<br>• Field research<br><br>Write "N/A" if the submission does not require an ethics statement.<br><br>General guidance is provided below. Consult the submission guidelines for detailed instructions. **Make sure that all information entered here is included in the Methods section of the manuscript.**	Ethics approval was obtained from the Jordanian Ministry of Health (MOH REC 160079).

Powered by Editorial Manager® and ProduXion Manager® from Aries Systems Corporation

**Format for specific study types**<br><br>**Human Subject Research (involving human participants and/or tissue)**<br>• Give the name of the institutional review board or ethics committee that approved the study<br>• Include the approval number and/or a statement indicating approval of this research<br>• Indicate the form of consent obtained (written/oral) or the reason that consent was not obtained (e.g. the data were analyzed anonymously)<br><br>**Animal Research (involving vertebrate animals, embryos or tissues)**<br>• Provide the name of the Institutional Animal Care and Use Committee (IACUC) or other relevant ethics board that reviewed the study protocol, and indicate whether they approved this research or granted a formal waiver of ethical approval<br>• Include an approval number if one was obtained<br>• If the study involved *non-human primates*, add *additional details* about animal welfare and steps taken to ameliorate suffering<br>• If anesthesia, euthanasia, or any kind of animal sacrifice is part of the study, include briefly which substances and/or methods were applied<br><br>**Field Research**<br><br>Include the following details if this study involves the collection of plant, animal, or other materials from a natural setting:<br>• Field permit number<br>• Name of the institution or relevant body that granted permission	
**Data Availability**<br><br>Authors are required to make all data underlying the findings described fully available, without restriction, and from the time of publication. PLOS allows rare exceptions to address legal and ethical concerns. See the PLOS Data Policy and FAQ for detailed information.	No - some restrictions will apply

A Data Availability Statement describing where the data can be found is required at submission. Your answers to this question constitute the Data Availability Statement and **will be published in the article**, if accepted.<br><br>**Important:** Stating 'data available on request from the author' is not sufficient. If your data are only available upon request, select 'No' for the first question and explain your exceptional situation in the text box.<br><br>Do the authors confirm that all data underlying the findings described in their manuscript are fully available without restriction?	
**Describe where the data may be found in full sentences. If you are copying our sample text, replace any instances of XXX with the appropriate details.**<br><br>• If the data are **held or will be held in a public repository**, include URLs, accession numbers or DOIs. If this information will only be available after acceptance, indicate this by ticking the box below. For example: *All XXX files are available from the XXX database (accession number(s) XXX, XXX.).*<br>• If the data are all contained **within the manuscript and/or Supporting Information files**, enter the following: *All relevant data are within the manuscript and its Supporting Information files.*<br>• If neither of these applies but you are able to provide **details of access elsewhere**, with or without limitations, please do so. For example:<br><br>*Data cannot be shared publicly because of [XXX]. Data are available from the XXX Institutional Data Access / Ethics Committee (contact via XXX) for researchers who meet the criteria for access to confidential data.*<br><br>*The data underlying the results presented in the study are available from (include the name of the third party*	All relevant data are within the manuscript and its Supporting Information files.

and contact information or URL).<br>• This text is appropriate if the data are owned by a third party and authors do not have permission to share the data.<br><br>* typeset	
Additional data availability information:	

Powered by Editorial Manager® and ProduXion Manager® from Aries Systems Corporation

Manuscript Click here to access/download;Manuscript;ResubmissionManuscript.Nov.do

Improving Syrian refugees' knowledge of medications and adherence following a randomized control trial assessing the effect of a medication management review service

Running title: Pharmacists improving Refugees' adherence and knowledge of their chronic medications

Registry: ClinicalTrials.gov Identifier: NCT04554810

Majdoleen Alalawneh. MSc
Graduate Teaching Research Assistant
College of Pharmacy, QU Health
Qatar University, PO Box 2713, Doha, Qatar
e-mail: malawneh@qu.edu.qa

Alberto Berardi. PhD
Associate Professor in Pharmaceutics,
Department of Pharmaceutical Sciences and Pharmaceutics,
Faculty of Pharmacy, Applied Science Private University, Amman 11931, Jordan
e-mail: a_berardi@asu.edu.jo

Nabeel Nuaimi. PhD
Professor in Clinical Pharmacy
Department of Pharmacy,
AlNoor University College,
Mosul, Iraq
e-mail: nabeel_nuaimi@yahoo.com.

Iman A. Basheti. PhD
Professor in Clinical Pharmacy
Department of Clinical Pharmacy and Therapeutics
Faculty of Pharmacy, Applied Sciences Private University, Amman, Jordan
Honorary Professor, Faculty of Pharmacy, The University of Sydney
e-mail: dr_iman@asu.edu.jo
Corresponding author: Iman A. Basheti. PhD
Email: dr_iman@asu.edu.jo Phone number - +962 79904800

Abstract:286

Manuscript:3239

Funding: The author(s) received no specific funding for this work.

Conflicts of interest: None.

Specific contributions of each contributor to the paper: All authors were involved in all parts of study and manuscript preparation including literature search, study design, analysis of data, manuscript preparation, and review of manuscript

Abstract

Background: Syrian refugees living in Jordan have many chroexacnic conditions and use many medications. Pharmacists delivering the Medication Management Review (MMR) service can have a role in improving this growing global refugees' problem.

Objectives: To assess the effect of the MMR service on adherence to treatment therapy and knowledge about chronic medications for Syrian refugees residing in Jordan.

Methods: This randomized intervention control single-blinded study was conducted in Jordan. Syrian refugees were recruited and randomized into intervention and control groups. Two home visits were delivered to each participant, at baseline and three months later. All participants completed questionnaires regarding adherence and knowledge. As a part of the MMR service, treatment-related problems (TRPs) were recognized for all patients; recommendations to resolve these TRPs were only delivered to intervention group refugees' physicians; TRPs were corrected. At follow-up, TRPs assessment, adherence and mediation knowledge were assessed for all refugee participants.

Results: Participants (n=106; intervention n=53, control n=53) had a number of medications and diagnosed chronic diseases of 5.8 ± 2.1 and 2.97 ± 1.16 per participant respectively. At follow-up, a significant decrease in the number of TRPs for refugees in the intervention group was found ((from 600 to 182, P <0.001), but not for control group (number stayed at 541 TRPs, P= 0.116). A significant improvement in the adherence and knowledge scores were noted in the intervention (P < 0.001 for both) but not the control group (P= 0.229, P= 0.07 respectively).

Conclusion: The MMR service can significantly improve refugees' TRPs, adherence to therapy and knowledge about chronic medications. If this approach was extended to the large scale, many refugees in need would be able to access a quality essential health-care service; a step towards achieving universal health coverage.

Keywords: Refugees, Medication management review, adherence, chronic medication, pharmaceutical care, Jordan, SDG 3, universal health coverage.

Introduction

The United Nations reported that, as of February 2016, a total of 13.5 million Syrian refugees had been identified as in need of humanitarian aid; of these refugees, 6.6 million were displaced within Syria and 4.8 million outside of Syria [1]. Refugees in general experience low living conditions, limited access to health care services as well as high poverty level in the country of settlement [1]. Taking these factors into consideration, refugees often have multiple health issues that need extensive health care [1-6]. In this context, low adherence to treatment and poor knowledge of medications and therapeutic regimens can be expected. Adherence to treatment can be an issue to many refugees, as getting their medications on time, and being provided with enough education on the importance of taking their medications as prescribed can be lacking[7, 8]. Medication knowledge has been defined as 'the awareness of the drug name, purpose, administration schedule, adverse effects or side-effects, or special administration instructions'[9]. It has been found that patients who need to take various medications are usually deficient in medication knowledge [10].

Globally, pharmaceutical care services have improved the use of medications and patients' adherence to their treatment [11]. In particular, patients' outcomes have been improved in various countries and in different populations and settings, through the use of the pharmacist-led Medication Management Review (MMR) service [12, 13]. This service is defined as "a distinct service or group of services that optimize clinical outcomes for each participant to ensure the appropriateness, effectiveness and safety for each participant's medication(s); in addition to ensuring the ability of the participant to take his/her medication(s) as should be" [14]. MMR services are designed to improve education of chronically ill patients, based on a comprehensive review of their treatment, past and current situation, in order to ultimately resolve adherence and medication knowledge issues [15, 16].

With the growing crises in Syria since 2011, large numbers of Syrian people have fled to other countries, including Jordan. The current "refugee crisis" has escalated sharply in Jordan and its impact on health and financial sections are widening [17]. In this scenario, Syrian refugees are facing many health-related problems, as their health care needs are not completely fulfilled [17]. Unfortunately, healthcare needs of Syrian people, based on their reported situation, remain still to be answered; thus, it is important to evaluate, and possibly address, refugees' healthcare needs in the countries where they settled in. No previous study, in Jordan or abroad, has assessed the needs and impact of the MMR service on refugees' adherence to their treatment therapy, and knowledge of their treatment.

The primary aim of this study was to assess refugees' adherence and knowledge of their chronic medications, and impact of the MMR service delivered by a clinical pharmacist on their adherence and knowledge of their chronic medications three months following delivering the service.

Method

Study design and clinical setting

This study was a randomized interventional clinical study, conducted over six months (May to October 2016) in different Jordanian cities, where most of Syrian refugees reside. The registration of this clinical study delayed, as it is not requested by Jordan (ClinicalTrials.gov Identifier: NCT04554810). Ethics approval was obtained from the Jordanian Ministry of Health (MOH REC 160079).

Clinics which are specialized for Syrian refugees were approached by the clinical pharmacist (researcher) in order to meet Syrian refugees, recruit eligible participants and arrange for their first home visits.

Inclusion criteria for the Syrian refugees included ≥ 18 years, living in Jordan for more than six months prior to study recruitment and intending to stay for the whole study period, having one chronic condition at least or taking 5 or more medications or taking more than 12 doses of a medication per day[18]. There was a deviation from the original study protocol regarding the sample size, as the calculated needed number of patients was 138 patients, however the recruited number was 109, due to time restriction.

An informed consent form was signed by all participants who accepted to participate. Eligible participants were then randomized into intervention and control groups using a predetermined list obtained by the computer randomization program (www.randomizer.org) before starting the study. The study was single – blinded, hence, participants were not informed of the group they were randomized into, but they were informed that they would have been in either of two study groups. The first group would have received the MMR service during the study period, while the to the other group directly after the study was completed (after three months' time).

Study Protocol

After the eligible patients were randomized into two groups, intervention group and control group, appointments were arranged at the physicians' clinics for all participants to be visited by the clinical pharmacist at their homes. At the baseline home visit, the clinical pharmacist documented participants' demographics, acute and chronic medical problems, history of present diseases, past medical history, lifestyle, family history, allergies, vital signs, physical examination information, diagnostic test data, lab results, current medications and issues related to the short and long term management of the patient .

The MMR service was completed following verification of collected baseline data. The home visits were planned not to exceed one hour, which is the usual time provided in related studies [19]. During these visits, self-completed questionnaires were completed by the participants, evaluating their adherence and knowledge about their chronic medications.

The clinical pharmacist identified and documented the treatment-related problems (TRPs) for each patient in both groups at baseline. All identified TRPs were supported by current therapeutic guidelines, reported in a letter format sent to the participant's physician. In the case any life-threatening TRPs were identified for control groups participants, they were excluded from the study for ethical considerations.

The physician was identified based on the participant's reported clinic and on participant's choice when more than one physician was visited by the participant. Following receipt of the letter, physicians addressed the recommendations and returned the letter to the pharmacist showing approved and rejected recommendations. Participants were called by the pharmacist to visit the physician and have the approved recommendations applied. Counselling and education were delivered to participants in the intervention group regarding their illnesses, knowledge of medications and adherence to their treatment.

Follow-up assessment

Three months post baseline, new appointments were arranged through a phone call by the clinical pharmacist, and all participants were revisited at home. Data needed to assess TRPs where recollected (as was done at baseline), plus the adherence and knowledge about chronic medications' questionnaires was completed for all participants. At the end of the study, control group participants received the MMR and pharmacist counselling service exactly as was delivered to the intervention group participants at baseline [20].

Data collection tools

For the purpose of data documentation and evaluation, the following self-completed questionnaires were used.

Adherence to medication

Adherence to medications was assessed by a developed and published questionnaire [21]. The questionnaire was composed of eight questions as follows: how often the patient during the last

month forgot to take his/her medication/s, skipped it, stopped it when feeling better, stopped it when feeling worse or stopped it when they experienced a side effect. The measurement scale used in this questionnaire was scored at 0 (never), 1 (rarely), 2 (sometimes), 3 (often) and 4 (always). Hence, adherence was analysed as a continuous scale out of 32. Higher scores indicted lower adherence by the patient.

Patients' limit of commitment to pharmacist advice and their main cause of non-adherence were also assessed by two questions at the end of this questionnaire. Lastly, patients were asked about the main reason behind their non-adherence to their medications.

For the intervention group, the clinical pharmacist answered any queries raised by the patients following their adherence assessment at baseline and study follow-up. However, for the control group, the clinical pharmacist did not answer any queries made by the patients following assessment, but for ethical reasons, all queries were recorded and answered after the end of the study.

Knowledge about chronic medications

Knowledge about chronic medications was evaluated using a validated and published questionnaire [21]. The questionnaire was modified by the research team to include close ended questions instead of open-ended question; this was done for the purpose of facilitating the understanding by the Syrian refugees. The questionnaire consisted of five questions related to patients' medications including 1- scientific medication name, 2- generic medication name, 3- how, 4- when, and 5- why was the patient taking each medication. The knowledge about medications was analysed as a continuous scale out of five. Higher scores indicated less knowledge about one's medication therapy.

Statistical analysis

Data were coded then entered into the Statistical Package for Social Sciences (SPSS), version 20. Continuous variables were expressed as mean ± SD. Differences within the same group

were detected using paired sample t-test for continuous variables. Group differences (between the intervention and control groups) were detected using the independent sample t-test or Mann Whitney U-test (based on the normality of data following testing) for continuous variables. Categorical data were expressed as proportion (%) and analysed using Chi-square test. A probability value of < 0.05 was considered to be statistically significant for all analysis's tests.

Sample size

Sample size calculation was based on the primary outcome variable, adherence to treatment, improvement before and following involvement in the MMR service. Depending on previous studies [15, 22], in order to detect a significant amendment in TRPs of 1 point difference [22], with a power of 85%, significance level of 5% and standard deviation of the change of 2.09 (variance based on the data from a previously published study[22]. The sample size of 138 was the needed required sample in both groups.

Results

A total of 123 Syrian refugee participants were approached for recruitment into the study. After participants' first meeting during the home visit by the clinical pharmacist, 116 participants (94.3%) were found eligible for study and 10 participants refused to continue in the study due to different causes (Figure 1). Participants (n=109) were randomized into intervention (n=55) and control (n=54) groups thereafter. The acceptance rate for home visits was 100%. No participants with life-threatening cases were identified in the control group.

After dropouts (3 patients), all participants (n= 106, 53 in each group) completed the study. Mean age of the participant was 58.5 ± 11.2, with about half being females (n= 51, 48.1%) and 51.9% had primary or preparatory educational level (Table 1).

The number (mean ± SD) of medications used per patient was 5.8 ± 2.1, and the number of chronic conditions per participant was 2.97 ± 1.16. Diabetes and hypertension were the most

common chronic conditions diagnosed among the participants (66.0%), followed by dyslipidemia (44.0%), cardiac illness (39.0%) and asthma (9.0%).

Adherence to medications at baseline and follow-up

A significant improvement in the adherence scores was noted in the active group across the study (P < 0.001, paired sample t-test). No significant improvement was found in the control group regarding the mean change in the adherence score (Figure 2).

With regards to the proportions of adherent patients, there was no significant difference between the intervention and the control groups at baseline (P = 0.54, Chi-square test) where 13.2% of patients in the intervention group were adherent to medications versus 11.3 % in the control group. Due to receiving the MMR service, a significant difference between the two groups was found at three months (P= 0.01, Chi-square test), where 32.1% of the patients in the intervention group became adherent compared to 9.4 % in the control group.

There was no significant difference with regards to patients' commitment to pharmacist advice between the two groups at baseline; at follow-up, a significant difference was found (P = 0.01, Chi-square test). Around 73% of intervention group patients were committed to the pharmacist advice at baseline compared to 88% commitment at follow-up.

Causes of non-adherence to medications were assessed at baseline visits. More than one third of the patients in the intervention group and the control group reported that the price was the main cause for non-adherence to their medication (39.7% and 37.7% respectively). Other causes for patients' non-adherence are listed in table (2). There was no statically significant difference between the two groups regarding the reported causes for non-adherence to medications at baseline (P= 0.764, Chi-square test).

Regarding the degree of patients' commitment to pharmacist advice, there was no significant difference between the two groups at baseline. At follow-up, a significant improvement was found in the intervention group but not the control group. (P = 0.01, Chi-square test) (Table 3).

Knowledge of medications at baseline and follow-up

At baseline, no significant difference was found between the intervention and the control groups with regards to ‘Knowledge about medications mean scores (P 0.499, Independent sample t-test). At follow-up, the difference was significant between the two groups ($P < 0.001$, Independent sample t-test). Across the study, there was a significant difference within the intervention group, but not the control group (Figure 3).

Upon comparison between the intervention and the control groups regarding the five domains included in the ‘knowledge about drug therapy’ questionnaire, there was no significant difference between the two groups in the five domains at baseline. However, significant differences resulted at follow-up regarding all domains (Table 4).

Discussion

The current study showed that the provision of the pharmacist-lead MMR service can lead to significant improvements in the adherence to medications and knowledge of medications for Syrian refugees living in Jordan. To our knowledge, this randomized controlled study is the first to be conducted for Syrian refugees around the world, assessing the impact of the MMR service on their adherence and knowledge about their chronic medications, decreasing the number of TRPs they suffer from. The study emphasizes the important role that clinical pharmacists can play in such crisis to help refugees to have better management of their health conditions and hence lives.

The Syrian conflict has been declared as a humanitarian crisis since 2011, leading to a health disaster for the country and for the region alike [23]. Overall, refugees are the weakest and most vulnerable group in the conflict setting, and therefore, their medical needs are expected to require urgent assistance [24]. Many studies conducted during the last years reported that pharmaceutical care interventions lead in most cases to desirable clinical and financial outcomes [25]. Pharmacists were found to help in improving patients’ health by reducing

medication-related side effects and encouraging medication adherence, decreasing physician visits, hospital admissions, and amending the whole primary care delivery [26]. But this study comes to show that the same can happen in a population of great need of this help, the Syrian refugees.

Adherence to medications is a critical part of general patient care. It is substantial for reaching the targeted goals for any patient, yet alone the refugees [27]. By opposition, non-adherence to medications is considered a main challenge to healthcare, with approximately 50% of patients reported not taking their chronic medications as prescribed 12 months after starting their therapy[28, 29]. The likely negative impact of non-adherence to medications on morbidity and mortality is well documented [30]. In the current study, adherence to medications was assessed utilizing a previously published questionnaire, self-reported by patients, allowing feasibility and ease of use by patients with different diseases, which is vital for the success of this assessment[31]. The changes in the non-adherence mean scores in the intervention group was significant, indicating the positive impact of the MMR service on participants' adherence to their medications. It is known from the literature that pharmacist interventions across the years, in its various forms, can improve participants' adherence. In Al-Eidan et al. (2002) for example, a prospective randomized study involving participants with Helicobacter pylori was found to significantly improve adherence of participants in the intervention group receiving a pharmacist counselling session compared to control group patients who did not receive the counselling session (92.1% versus 23.7%) [32]. Jarab et al. conducted a study in 2012 involving diabetic patients in Jordan showing that the pharmaceutical intervention delivered in that study resulted in significantly lower proportion of non-adherent participants in the intervention group (28.6%) compared with the control group participants (64.6%)[33]. Moreover, Brummel & Carlson conducted a study in 2016 revealing that the comprehensive medication management (CMM) service among USA participants resulted in significant

improvement in their medication adherence[30]. Basheti et al. conducted a study in 2016 as well, showing that the MMR service resulted in a significant reduction of non-adherence scores among the intervention group participants[15]. Such findings support the results of the current study, where adherence to medications was improved significantly as a result of pharmaceutical care services such as the MMR service.

Knowledge of medications is essential for proper adherence and good management of chronic conditions to be reached. Participants' knowledge about their medications showed a significant improvement across the study for the intervention group, but not the control group. A previous study showed that a significant improvement in the knowledge about medications was seen for participants in the intervention group as a result of a pharmacist-led intervention [34]. In addition, a large number of American studies (n= 298) included in a meta-analysis review proved that 57.1% of these studies favour the positive impact of pharmacists on the participants' knowledge about their medications [35].

The approach described here, i.e. wherein the MMR service is delivered to a population (the refugees) who needs it and can benefit from it the most, represents a virtuous strategy to target and maximize the impact of health care professional services. The extension of this model to large scale would sharply address and support some of the targets of the Sustainable Development Goal (SDG) 3 "*Ensure healthy lives and promote well-being for all at all ages*". In particular, delivery of MMR services to refuges would contribute to achieve the SGD targets 3.4 "*By 2030, reduce by one third premature mortality from non-communicable diseases through prevention and treatment and promote mental health and well-being*" and 3.8 "*Achieve universal health coverage, including financial risk protection, access to quality essential health-care services and access to safe, effective, quality and affordable essential medicines and vaccines for all". This comes in line with the WHO recommendations [36] supporting the execution of the recently published report by the High-Level Commission on Health

Employment and Economic Growth [37] calling for "ambitious solutions to ensure that the world has the right number of jobs for health workers with the right skills and in the right places to deliver universal health coverage". A focus on trained universal health-workforce that has the ability to deliver different healthcare services in humanitarian settings was highlighted [37].

Study limitations

A longer follow up could have added new insights to the results. We did not assess health literacy abound the current medications used by patients which could have further explained improvements in medication knowledge improvements. We did not study recommendations rejected by the physicians nor the ones not applied by the patients. This would be important for future studies to assess the value of physician-patient involvement in decision making [43].

Conclusion

This study revealed that many refugees with numerous medications and chronic health condition have numerous TRPs, low adherence and low knowledge of their medications. Significant improvements in adherence and knowledge to treatment was reached as a result of the MMR service provided by the clinical pharmacist. Pharmaceutical care is an essential aspect of patient care and communication between healthcare providers can help enhance this care with expressively upgraded consequences for the individual refugee patient. While this understanding is intuitively accurate, few studies have apprehended the findings to report these benefits. Therefore, this study provides an important contribution to this understandings of medication management for displaced refugee communities. The positive outcomes of this study emphasizes the important role pharmacists can play in conveying a vital and required service in a vast humanitarian setting globally.

References

1. Doganay M, Demiraslan H. Refugees of the Syrian civil war: impact on reemerging infections, health services, and biosecurity in Turkey. Health security. 2016;14(4):220-5.
2. Guendelman S, Schauffler HH, Pearl M. Unfriendly shores: how immigrant children fare in the US health system. Health Affairs. 2001;20(1):257-66.
3. Crockett M. New faces from faraway places: Immigrant child health in Canada. Paediatrics & child health. 2005;10(5):277-81.
4. Sanmartin C, Ross N. Experiencing difficulties accessing first-contact health services in Canada: Canadians without regular doctors and recent immigrants have difficulties accessing first-contact healthcare services. Reports of difficulties in accessing care vary by age, sex and region. Healthcare Policy. 2006;1(2):103-19.
5. Pottie K, Greenaway C, Feightner J, Welch V, Swinkels H, Rashid M, et al. Evidence-based clinical guidelines for immigrants and refugees. Cmaj. 2011;183(12):E824-E925.
6. Doocy S, Lyles E, Roberton T, Akhu-Zaheya L, Oweis A, Burnham G. Prevalence and care-seeking for chronic diseases among Syrian refugees in Jordan. BMC Public Health. 2015;15(1):1097-2106.
7. Bailey C, Kodack M. Patient adherence to medication requirements for therapy of type 2 diabetes. International journal of clinical practice. 2011;65(3):314-22.
8. Basheti IA, El Hait SS, Qunaibi EA, Aburuz S, Bulatova N. Associations between patient factors and medication adherence: A Jordanian experience. Pharmacy Practice (Granada). 2016;14(1):1-7.
9. Ascione FJ, Kirscht JP, Shimp LA. An assessment of different components of patient medication knowledge. Medical care. 1986:1018-28.
10. Williford SL, Johnson DF. Impact of pharmacist counseling on medication knowledge and compliance. Military medicine. 1995;160(11):561-4.
11. Mahdikhani S, Dabaghzadeh F. Benefits of Pharmacist's Participation on Hospitalist Team. Acta Medica Iranica. 2016;54(2):140-5.
12. Ramalho de Oliveira D, Brummel AR, Miller DB. Medication therapy management: 10 years of experience in a large integrated health care system. Journal of Managed Care Pharmacy. 2010;16(3):185-95.
13. Castelino RL, Bajorek BV, Chen TF. Are interventions recommended by pharmacists during Home Medicines Review evidence-based? Journal of evaluation in clinical practice. 2011;17(1):104-10.
14. Bluml BM. Definition of medication therapy management: development of professionwide consensus. Journal of the American Pharmacists Association. 2005;45(5):566-72.
15. Basheti IA, Al-Qudah RA, Obeidat NM, Bulatova NR. Home medication management review in outpatients with chronic diseases in Jordan: a randomized control trial. International journal of clinical pharmacy. 2016;38(2):404-13.
16. Saleem F, Hassali MA, Shafie AA, Ul Haq N, Farooqui M, Aljadhay H, et al. Pharmacist intervention in improving hypertension-related knowledge, treatment medication adherence and health-related quality of life: a non-clinical randomized controlled trial. Health Expectations. 2015;18(5):1270-81.
17. Sirin SR, Rogers-Sirin L. The educational and mental health needs of Syrian refugee children: Migration Policy Institute Washington, DC; 2015, Acced by: http://wellnessresources.pbworks.com/w/file/fetch/117239565/Education%20and%20Mental%20Health%20Needs%20of%20Syrian%20Refugee%20Children.pdf.

18. Council APA. Guidelines for medication management in residential aged care facilities. Accessed by: https://www.health.gov.au/sites/default/files/documents/2020/02/guiding-principles-for-medication-management-in-residential-aged-care-facilities.pdf. 2007:1-98.

19. Freeman CR, Cottrell WN, Kyle G, Williams ID, Nissen L. An evaluation of medication review reports across different settings. International journal of clinical pharmacy. 2013;35(1):5-13.

20. AbuRuz SM, Bulatova NR, Yousef A-MM, Al-Ghazawi MA, Alawwa IA, Al-Saleh A. Comprehensive assessment of treatment related problems in hospitalized medicine patients in Jordan. International journal of clinical pharmacy. 2011;33(3):501-11.

21. Al-Qudah RA, Bulatova NR, Obeidatc NM, Basheti IA. Impact of home medication management review on medication adherence among Jordanian patients. Journal of Pharmaceutical Health Services Research. 2018;[Accepted].

22. Coutts A, Fouad FM. Response to Syria's health crisis—poor and uncoordinated. The Lancet. 2013;381(9885):2242-3.

23. El-Khatib Z, Scales D, Vearey J, Forsberg BC. Syrian refugees, between rocky crisis in Syria and hard inaccessibility to healthcare services in Lebanon and Jordan. Conflict and health. 2013;7(1):18.

24. Abughosh SM, Wang X, Serna O, Henges C, Masilamani S, James Essien E, et al. A pharmacist telephone intervention to identify adherence barriers and improve adherence among nonadherent patients with comorbid hypertension and diabetes in a Medicare Advantage Plan. Journal of managed care & specialty pharmacy. 2016;22(1):63-73.

25. Mossialos E, Courtin E, Naci H, Benrimoj S, Bouvy M, Farris K, et al. From “retailers” to health care providers: transforming the role of community pharmacists in chronic disease management. Health Policy. 2015;119(5):628-39.

26. Lam WY, Fresco P. Medication adherence measures: an overview. BioMed research international. 2015:1-12.

27. Krousel-Wood M, Elizabeth H, Joyce C, Rachael R, Dornelles A, Webber LS, et al. Differences in cardiovascular disease risk when antihypertensive medication adherence is assessed by pharmacy fill versus self-report: the Cohort Study of Medication Adherence among Older Adults (CoSMO). Journal of hypertension. 2015;33(2):412-21.

28. Pellegrino AN, Martin MT, Tilton JJ, Touchette DR. Medication therapy management services. Drugs. 2009;69(4):393-406.

29. Brummel A, Carlson AM. Comprehensive medication management and medication adherence for chronic conditions. Journal of managed care & specialty pharmacy. 2016;22(1):56-62.

30. Tan X, Patel I, Chang J. Review of the four item Morisky medication adherence scale (MMAS-4) and eight item Morisky medication adherence scale (MMAS-8). INNOVATIONS in pharmacy. 2014;5(3):5-35.

31. Al-Eidan F, McElnay J, Scott M, McConnell J. Management of Helicobacter pylori eradication–the influence of structured counselling and follow-up. British journal of clinical pharmacology. 2002;53(2):163-71.

32. Jarab AS, Alqudah SG, Mukattash TL, Shattat G, Al-Qirim T. Randomized controlled trial of clinical pharmacy management of patients with type 2 diabetes in an outpatient diabetes clinic in Jordan. Journal of Managed Care Pharmacy. 2012;18(7):516-26.

33. Chow EP, Hassali MA, Saleem F, Aljadhey H. Effects of pharmacist-led patient education on diabetes-related knowledge and medication adherence: a home-based study. Health Education Journal. 2016;75(4):421-33.

34. Chisholm-Burns MA, Lee JK, Spivey CA, Slack M, Herrier RN, Hall-Lipsy E, et al. US pharmacists' effect as team members on patient care: systematic review and meta-analyses. Medical care. 2010:923-33.

35. World Health Organization. Seventieth World Health Assembly. Provisional agenda item 13.1. Human resources for health and implementation of the outcomes of the United Nations’ High Level Commission on Health Employment and Economic Growth. World Health Assembly Journal

Available at 18 http://appswhoint/gb/ebwha/pdf_files/WHA70'/A70_18-enpdf?ua=1 Accessed 4 February 2018. 2017.
36. World Health Organization. High-Level Commission on Health Employment and Economic Growth. Working for health and growth: investing in the health workforce. Report of the High-Level Commission on Health Employment and Economic Growth. Found at http://apps.who.int/iris/bitstream/10665/250047/1/9789241511308-eng.pdf. Accesssed on 4 February 2018. . 2016.

Figure legend

Figure (1). Consort diagram showing patients' recruitment and retention during the study period

Figure (2). 'Adherence to medication' mean changes at baseline and follow-up

Figure (3). The knowledge about drug therapy mean changes at baseline and follow-up

Tables

Table 1. Demographic characteristics of the study sample.

Parameter	Total number of patients	Intervention group	Control group	P-value
**Number of patients, n (%)**	106 (100)	53 (50)	53 (50)	--------
**Age** (year),**mean (SD)**	58.5 (11.2)	60.1 (12.1)	56.9 (10.1)	0.157[1]
**Gender n (%)**				
Male	55 (51.9)	26 (49.1)	29 (54.7)	0.560[2]
Female	51 (48.1)	27 (50.9)	24 (45.3)	
**BMI mean (SD)**	30.24 (7.1)	30.3 (7.02)	30.2 (7.3)	0.813[3]
**Body mass category,(n) %**				
Under weight	4 (3.8)	3 (5.7)	1 (1.9)	
Normal	16 (15.1)	9 (17.0)	7 (13.2)	
Over weight	32 (30.2)	12 (22.6)	20 (37.7)	0.469[2]
Obese class 1	36 (33.9)	18 (34)	18 (34)	
Obese class 2	12 (11.3)	8 (15.1)	4 (7.5)	
Morbid obesity	6 (5.7)	3 (5.6)	3 (5.7)	
**Marital status, n (%)**				/
Single	3 (2.8)	2 (3.8)	1 (1.9)	
Married	83 (78.3)	39 (73.6)	44 (83.0)	0.488[2]
Widow	20 (18.9)	12 (22.6)	8 (15.1)	
**Exercise, n (%)**				
Yes	30 (28.3)	13 (24.5)	17 (32.1)	0.388[2]
No	76 (71.7)	40 (75.5)	36 (67.9)	
**Smoking, n (%)**				
Yes	44 (41.5)	20 (37.7)	24 (45.3)	0.430[2]
No	62 (58.5)	33 (62.3)	29 (54.7)	
**Caffeine, n (%)**				
Yes	81 (76.4)	41 (77.4)	40 (75.5)	0.819[2]
No	25 (23.6)	12 (22.6)	13 (24.5)	
**Education level, n (%)**				
Primary + preparatory	55 (51.9)	30 (56.6)	25 (47.2)	
High school	29 (27.4)	16 (30.2)	13 (24.5)	0.299[2]
BSc	19 (17.9)	6 (11.3)	13 (24.5)	
MSc	3 (2.8)	1 (1.9)	2 (3.8)	
**Clinic, n (%)**				
Emirate Red Crescent	40 (37.7)	20 (37.7)	20 (37.7)	
Jordan Health AID Society	32 (30.2)	14 (26.4)	18 (34.0)	
Private	30 (28.3)	16 (30.2)	14 (26.4)	0.742[2]
Social charity	3 (2.8)	2 (3.8)	1 (1.9)	
Governmental center	1 (0.9)	1 (1.9)	0 (0.0)	

n: Number of patients **SD**: Standard deviation
1 Analysis by independent-sample t-test **2** Analysis by chi-square test

3 Analysis by Mann Whitney U-te

Table 2. Main causes for non-adherence to medications at baseline

Causes of non-adherence, n(%)	Intervention group (n=53)	Control group (n=53)
**Price**	21 (39.7)	20 (37.7)
**Timing of dose**	1 (1.9)	2 (3.9)
**Forgetting**	7 (13.2)	8 (15.1)
**I do not like medicines**	4 (7.5)	8 (15.1)
**Medicine does not work**	4 (7.5)	5 (9.4)
**Side effects**	9 (17)	5 (9.4)
**High number of pills every day**	7 (13.2)	5 (9.4)

n: Number of patients

Table 3. Commitment to pharmacist advice at baseline and follow-up

Parameter	Intervention group n=53		Control group n=53		P-value
**Commitment to pharmacist advice at baseline, n (%)**	Never	0 (0.0)	Never	1 (1.9)	0.495[1]
	Rare	4 (7.5)	Rare	7 (13.2)	
	Sometimes	10 (18.9)	Sometimes	10 (18.9)	
	Usually	17 (32.1)	Usually	20 (37.7)	
	Always	22 (41.5)	Always	15 (28.3)	
**Commitment to pharmacist advice at follow-up, n (%)**	Never	0 (0.0)	Never	1 (1.9)	0.010[1]
	Rare	0 (0.0)	Rare	6 (11.3)	
	Sometimes	6 (11.3)	Sometimes	13 (24.5)	
	Usually	21 (39.6)	Usually	19 (35.8)	
	Always	26 (49.1)	Always	14 (26.5)	

n: Number of patients **1** Analysis by Chi-square test

Table 4.Comparison of the 'knowledge about drug therapy' five domains between the two groups.

Parameter n (%)	Intervention group n=53		Control group n=53		P-value	P-value
	Baseline	Follow-up	Baseline	Follow-up	Baseline	Follow-up
**Know the scientific name of medication**					0.6[1]	< 0.001[1]
• I know very well	0 (0.0)	5 (9.4)	0 (0.0)	0 (0.0)		
• I know to some extent	2 (3.8)	14 (26.4)	5 (9.4)	5 (9.4)		
• I know the name for some of my medications only	10 (18.9)	15 (28.3)	7 (13.2)	8 (15.1)		
• I don't know	15 (28.3)	11 (20.8)	16 (30.2)	16 (30.2)		
• I don't know at all	26 (49.1)	8 (15.1)	25 (47.2)	24 (45.3)		
**Know the generic name of medication**					0.961[1]	< 0.001[1]
• I know very well	0 (0.0)	12 (22.6)	0 (0.0)	0 (0.0)		
• I know to some extent	9 (17)	19 (35.8)	9 (17)	9 (17)		
• I know the names for some of my medications only	17 (32.1)	16 (30.2)	17 (32.1)	18 (34)		
• I don't know	14 (26.4)	6 (11.3)	12 (22.6)	13 (24.5)		
• I don't know at all	13 (24.5)	0 (0.0)	15 (28.3)	13 (24.5)		
**Know how to take the medication**					0.092[1]	< 0.001[1]
• I know very well	9 (17)	37 (69.8)	9 (17)	9 (17)		
• I know to some extent	23 (43.4)	11 (20.8)	21 (39.6)	21 (39.6)		
• I know how to take some of my medications only	14 (26.4)	5 (9.4)	16 (30.2)	16 (30.2)		
• I don't know	2 (3.8)	0 (0.0)	7 (13.2)	7 (13.2)		
• I don't know at all	5 (9.4)	0 (0.0)	0 (0.0)	0 (0.0)		
**Know when to take the medication**					0.446[1]	< 0.001[1]
• I know very well	7 (13.2)	41 (77.4)	8 (15.1)	8 (15.1)		
• I know to some extent	24 (45.3)	10 (18.9)	23 (43.4)	24 (45.3)		
• I know when to take some of my medications only	13 (24.5)	2 (3.8)	18 (34)	17 (32.1)		
• I don't know	7 (13.2)	0 (0.0)	4 (7.5)	4 (7.5)		
• I don't know at all	2 (3.8)	0 (0.0)	0 (0.0)	0 (0.0)		

**Know why taking the medication**						
• **I know very well**	2 (3.8)	32 (60.4)	4 (7.5)	4 (7.5)		
• **I know to some extent**	11 (20.8)	15 (28.3)	12 (22.6)	11 (20.8)		
• **I know why I take some of my medications only**	29 (54.7)	6 (11.3)	31 (58.5)	34 (64.2)	0.398[1]	< 0.001[1]
• **I don't know**	8 (15.1)	0 (0.0)	6 (11.3)	4 (7.5)		
• **I don't know at all**	3 (5.7)	0 (0.0)	0 (0.0)	0 (0.0)		

n: Number of patients
1 Analysis by Chi-square test

1. Doganay M, Demiraslan H. Refugees of the Syrian civil war: impact on reemerging infections, health services, and biosecurity in Turkey. Health security. 2016;14(4):220-5.
2. Guendelman S, Schauffler HH, Pearl M. Unfriendly shores: how immigrant children fare in the US health system. Health Affairs. 2001;20(1):257-66.
3. Crockett M. New faces from faraway places: Immigrant child health in Canada. Paediatrics & child health. 2005;10(5):277-81.
4. Sanmartin C, Ross N. Experiencing difficulties accessing first-contact health services in Canada: Canadians without regular doctors and recent immigrants have difficulties accessing first-contact healthcare services. Reports of difficulties in accessing care vary by age, sex and region. Healthcare Policy. 2006;1(2):103-19.
5. Pottie K, Greenaway C, Feightner J, Welch V, Swinkels H, Rashid M, et al. Evidence-based clinical guidelines for immigrants and refugees. Cmaj. 2011;183(12):E824-E925.
6. Doocy S, Lyles E, Roberton T, Akhu-Zaheya L, Oweis A, Burnham G. Prevalence and care-seeking for chronic diseases among Syrian refugees in Jordan. BMC Public Health. 2015;15(1):1097-2106.
7. Bailey C, Kodack M. Patient adherence to medication requirements for therapy of type 2 diabetes. International journal of clinical practice. 2011;65(3):314-22.
8. Basheti IA, El Hait SS, Qunaibi EA, Aburuz S, Bulatova N. Associations between patient factors and medication adherence: A Jordanian experience. Pharmacy Practice (Granada). 2016;14(1):1-7.
9. Ascione FJ, Kirscht JP, Shimp LA. An assessment of different components of patient medication knowledge. Medical care. 1986:1018-28.
10. Williford SL, Johnson DF. Impact of pharmacist counseling on medication knowledge and compliance. Military medicine. 1995;160(11):561-4.
11. Mahdikhani S, Dabaghzadeh F. Benefits of Pharmacist's Participation on Hospitalist Team. Acta Medica Iranica. 2016;54(2):140-5.
12. Ramalho de Oliveira D, Brummel AR, Miller DB. Medication therapy management: 10 years of experience in a large integrated health care system. Journal of Managed Care Pharmacy. 2010;16(3):185-95.
13. Castelino RL, Bajorek BV, Chen TF. Are interventions recommended by pharmacists during Home Medicines Review evidence-based? Journal of evaluation in clinical practice. 2011;17(1):104-10.
14. Bluml BM. Definition of medication therapy management: development of professionwide consensus. Journal of the American Pharmacists Association. 2005;45(5):566-72.
15. Basheti IA, Al-Qudah RA, Obeidat NM, Bulatova NR. Home medication management review in outpatients with chronic diseases in Jordan: a randomized control trial. International journal of clinical pharmacy. 2016;38(2):404-13.
16. Saleem F, Hassali MA, Shafie AA, Ul Haq N, Farooqui M, Aljadhay H, et al. Pharmacist intervention in improving hypertension-related knowledge, treatment medication adherence and health-related quality of life: a non-clinical randomized controlled trial. Health Expectations. 2015;18(5):1270-81.
17. Sirin SR, Rogers-Sirin L. The educational and mental health needs of Syrian refugee children: Migration Policy Institute Washington, DC; 2015, Acced by: http://wellnessresources.pbworks.com/w/file/fetch/117239565/Education%20and%20Mental%20Health%20Needs%20of%20Syrian%20Refugee%20Children.pdf.
18. Council APA. Guidelines for medication management in residential aged care facilities. Accessed by: https://www.health.gov.au/sites/default/files/documents/2020/02/guiding-principles-for-medication-management-in-residential-aged-care-facilities.pdf. 2007:1-98.
19. Freeman CR, Cottrell WN, Kyle G, Williams ID, Nissen L. An evaluation of medication review reports across different settings. International journal of clinical pharmacy. 2013;35(1):5-13.

20. Al Alawneh M, Nuaimi N, Basheti IA. Pharmacists in humanitarian crisis settings: Assessing the impact of pharmacist-delivered home medication management review service to Syrian refugees in Jordan. Research in social & administrative pharmacy : RSAP. 2019;15(2):164-72.
21. AbuRuz SM, Bulatova NR, Yousef A-MM, Al-Ghazawi MA, Alawwa IA, Al-Saleh A. Comprehensive assessment of treatment related problems in hospitalized medicine patients in Jordan. International journal of clinical pharmacy. 2011;33(3):501-11.
22. Al-Qudah RA, Bulatova NR, Obeidatc NM, Basheti IA. Impact of home medication management review on medication adherence among Jordanian patients. Journal of Pharmaceutical Health Services Research. 2018;[Accepted].
23. Coutts A, Fouad FM. Response to Syria's health crisis—poor and uncoordinated. The Lancet. 2013;381(9885):2242-3.
24. El-Khatib Z, Scales D, Vearey J, Forsberg BC. Syrian refugees, between rocky crisis in Syria and hard inaccessibility to healthcare services in Lebanon and Jordan. Conflict and health. 2013;7(1):18.
25. Abughosh SM, Wang X, Serna O, Henges C, Masilamani S, James Essien E, et al. A pharmacist telephone intervention to identify adherence barriers and improve adherence among nonadherent patients with comorbid hypertension and diabetes in a Medicare Advantage Plan. Journal of managed care & specialty pharmacy. 2016;22(1):63-73.
26. Mossialos E, Courtin E, Naci H, Benrimoj S, Bouvy M, Farris K, et al. From "retailers" to health care providers: transforming the role of community pharmacists in chronic disease management. Health Policy. 2015;119(5):628-39.
27. Lam WY, Fresco P. Medication adherence measures: an overview. BioMed research international. 2015:1-12.
28. Krousel-Wood M, Elizabeth H, Joyce C, Rachael R, Dornelles A, Webber LS, et al. Differences in cardiovascular disease risk when antihypertensive medication adherence is assessed by pharmacy fill versus self-report: the Cohort Study of Medication Adherence among Older Adults (CoSMO). Journal of hypertension. 2015;33(2):412-21.
29. Pellegrino AN, Martin MT, Tilton JJ, Touchette DR. Medication therapy management services. Drugs. 2009;69(4):393-406.
30. Brummel A, Carlson AM. Comprehensive medication management and medication adherence for chronic conditions. Journal of managed care & specialty pharmacy. 2016;22(1):56-62.
31. Tan X, Patel I, Chang J. Review of the four item Morisky medication adherence scale (MMAS-4) and eight item Morisky medication adherence scale (MMAS-8). INNOVATIONS in pharmacy. 2014;5(3):5-35.
32. Al-Eidan F, McElnay J, Scott M, McConnell J. Management of Helicobacter pylori eradication–the influence of structured counselling and follow-up. British journal of clinical pharmacology. 2002;53(2):163-71.
33. Jarab AS, Alqudah SG, Mukattash TL, Shattat G, Al-Qirim T. Randomized controlled trial of clinical pharmacy management of patients with type 2 diabetes in an outpatient diabetes clinic in Jordan. Journal of Managed Care Pharmacy. 2012;18(7):516-26.
34. Chow EP, Hassali MA, Saleem F, Aljadhey H. Effects of pharmacist-led patient education on diabetes-related knowledge and medication adherence: a home-based study. Health Education Journal. 2016;75(4):421-33.
35. Chisholm-Burns MA, Lee JK, Spivey CA, Slack M, Herrier RN, Hall-Lipsy E, et al. US pharmacists' effect as team members on patient care: systematic review and meta-analyses. Medical care. 2010:923-33.
36. World Health Organization. Seventieth World Health Assembly. Provisional agenda item 13.1. Human resources for health and implementation of the outcomes of the United Nations' High Level Commission on Health Employment and Economic Growth. World Health Assembly Journal Available at 18 http://appswhoint/gb/ebwha/pdf_files/WHA70'/A70_18-enpdf?ua=1 Accessed 4 February 2018. 2017.

37. World Health Organization. High-Level Commission on Health Employment and Economic Growth. Working for health and growth: investing in the health workforce. Report of the High-Level Commission on Health Employment and Economic Growth. Found at http://apps.who.int/iris/bitstream/10665/250047/1/9789241511308-eng.pdf. Accesssed on 4 February 2018. . 2016.

1. Doganay M, Demiraslan H. Refugees of the Syrian civil war: impact on reemerging infections, health services, and biosecurity in Turkey. Health security. 2016;14(4):220-5.
2. Guendelman S, Schauffler HH, Pearl M. Unfriendly shores: how immigrant children fare in the US health system. Health Affairs. 2001;20(1):257-66.
3. Crockett M. New faces from faraway places: Immigrant child health in Canada. Paediatrics & child health. 2005;10(5):277-81.
4. Sanmartin C, Ross N. Experiencing difficulties accessing first-contact health services in Canada: Canadians without regular doctors and recent immigrants have difficulties accessing first-contact healthcare services. Reports of difficulties in accessing care vary by age, sex and region. Healthcare Policy. 2006;1(2):103.
5. Pottie K, Greenaway C, Feightner J, Welch V, Swinkels H, Rashid M, et al. Evidence-based clinical guidelines for immigrants and refugees. Cmaj. 2011;183(12):E824-E925.
6. Doocy S, Lyles E, Roberton T, Akhu-Zaheya L, Oweis A, Burnham G. Prevalence and care-seeking for chronic diseases among Syrian refugees in Jordan. BMC Public Health. 2015;15(1):1097-2106.
7. Bailey C, Kodack M. Patient adherence to medication requirements for therapy of type 2 diabetes. International journal of clinical practice. 2011;65(3):314-22.
8. Basheti IA, El Hait SS, Qunaibi EA, Aburuz S, Bulatova N. Associations between patient factors and medication adherence: A Jordanian experience. Pharmacy Practice (Granada). 2016;14(1):1-7.
9. Ascione FJ, Kirscht JP, Shimp LA. An assessment of different components of patient medication knowledge. Medical care. 1986:1018-28.
10. Williford SL, Johnson DF. Impact of pharmacist counseling on medication knowledge and compliance. Military medicine. 1995;160(11):561-4.
11. Mahdikhani S, Dabaghzadeh F. Benefits of Pharmacist's Participation on Hospitalist Team. Acta Medica Iranica. 2016;54(2):140-5.
12. Ramalho de Oliveira D, Brummel AR, Miller DB. Medication therapy management: 10 years of experience in a large integrated health care system. Journal of Managed Care Pharmacy. 2010;16(3):185-95.
13. Castelino RL, Bajorek BV, Chen TF. Are interventions recommended by pharmacists during Home Medicines Review evidence-based? Journal of evaluation in clinical practice. 2011;17(1):104-10.
14. Bluml BM. Definition of medication therapy management: development of professionwide consensus. Journal of the American Pharmacists Association. 2005;45(5):566-72.
15. Basheti IA, Al-Qudah RA, Obeidat NM, Bulatova NR. Home medication management review in outpatients with chronic diseases in Jordan: a randomized control trial. International journal of clinical pharmacy. 2016;38(2):404-13.
16. Saleem F, Hassali MA, Shafie AA, Ul Haq N, Farooqui M, Aljadhay H, et al. Pharmacist intervention in improving hypertension-related knowledge, treatment medication adherence and health-related quality of life: a non-clinical randomized controlled trial. Health Expectations. 2015;18(5):1270-81.
17. Sirin SR, Rogers-Sirin L. The educational and mental health needs of Syrian refugee children: Migration Policy Institute Washington, DC; 2015.
18. Council APA. Guidelines for medication management in residential aged care facilities. Accessed by: https://www.health.gov.au/sites/default/files/documents/2020/02/guiding-principles-for-medication-management-in-residential-aged-care-facilities.pdf. 2007:1-98.

19. Freeman CR, Cottrell WN, Kyle G, Williams ID, Nissen L. An evaluation of medication review reports across different settings. International journal of clinical pharmacy. 2013;35(1):5-13.
20. Al Alawneh M, Nuaimi N, Basheti IA. Pharmacists in humanitarian crisis settings: Assessing the impact of pharmacist-delivered home medication management review service to Syrian refugees in Jordan. Research in social & administrative pharmacy : RSAP. 2019;15(2):164-72.
21. AbuRuz SM, Bulatova NR, Yousef A-MM, Al-Ghazawi MA, Alawwa IA, Al-Saleh A. Comprehensive assessment of treatment related problems in hospitalized medicine patients in Jordan. International journal of clinical pharmacy. 2011;33(3):501-11.
22. Al-Qudah RA, Bulatova NR, Obeidatc NM, Basheti IA. Impact of home medication management review on medication adherence among Jordanian patients. Journal of Pharmaceutical Health Services Research. 2018;[Accepted].
23. Coutts A, Fouad FM. Response to Syria's health crisis—poor and uncoordinated. The Lancet. 2013;381(9885):2242-3.
24. El-Khatib Z, Scales D, Vearey J, Forsberg BC. Syrian refugees, between rocky crisis in Syria and hard inaccessibility to healthcare services in Lebanon and Jordan. Conflict and health. 2013;7(1):18.
25. Abughosh SM, Wang X, Serna O, Henges C, Masilamani S, James Essien E, et al. A pharmacist telephone intervention to identify adherence barriers and improve adherence among nonadherent patients with comorbid hypertension and diabetes in a Medicare Advantage Plan. Journal of managed care & specialty pharmacy. 2016;22(1):63-73.
26. Mossialos E, Courtin E, Naci H, Benrimoj S, Bouvy M, Farris K, et al. From “retailers” to health care providers: transforming the role of community pharmacists in chronic disease management. Health Policy. 2015;119(5):628-39.
27. Lam WY, Fresco P. Medication adherence measures: an overview. BioMed research international. 2015:1-12.
28. Krousel-Wood M, Elizabeth H, Joyce C, Rachael R, Dornelles A, Webber LS, et al. Differences in cardiovascular disease risk when antihypertensive medication adherence is assessed by pharmacy fill versus self-report: the Cohort Study of Medication Adherence among Older Adults (CoSMO). Journal of hypertension. 2015;33(2):412.
29. Pellegrino AN, Martin MT, Tilton JJ, Touchette DR. Medication therapy management services. Drugs. 2009;69(4):393-406.
30. Brummel A, Carlson AM. Comprehensive medication management and medication adherence for chronic conditions. Journal of managed care & specialty pharmacy. 2016;22(1):56-62.
31. Tan X, Patel I, Chang J. Review of the four item Morisky medication adherence scale (MMAS-4) and eight item Morisky medication adherence scale (MMAS-8). INNOVATIONS in pharmacy. 2014;5(3):5.
32. Al-Eidan F, McElnay J, Scott M, McConnell J. Management of Helicobacter pylori eradication–the influence of structured counselling and follow-up. British journal of clinical pharmacology. 2002;53(2):163-71.
33. Jarab AS, Alqudah SG, Mukattash TL, Shattat G, Al-Qirim T. Randomized controlled trial of clinical pharmacy management of patients with type 2 diabetes in an outpatient diabetes clinic in Jordan. Journal of Managed Care Pharmacy. 2012;18(7):516-26.
34. Chow EP, Hassali MA, Saleem F, Aljadhey H. Effects of pharmacist-led patient education on diabetes-related knowledge and medication adherence: a home-based study. Health Education Journal. 2016;75(4):421-33.
35. Chisholm-Burns MA, Lee JK, Spivey CA, Slack M, Herrier RN, Hall-Lipsy E, et al. US pharmacists' effect as team members on patient care: systematic review and meta-analyses. Medical care. 2010:923-33.
36. World Health Organization. Seventieth World Health Assembly. Provisional agenda item 13.1. Human resources for health and implementation of the outcomes of the United Nations’ High Level Commission on Health Employment and Economic Growth. World Health Assembly Journal

Available at 18 http://appswhoint/gb/ebwha/pdf_files/WHA70'/A70_18-enpdf?ua=1 Accessed 4 February 2018. 2017.
37. World Health Organization. High-Level Commission on Health Employment and Economic Growth. Working for health and growth: investing in the health workforce. Report of the High-Level Commission on Health Employment and Economic Growth. Found at http://apps.who.int/iris/bitstream/10665/250047/1/9789241511308-eng.pdf. Accesssed on 4 February 2018. . 2016.

1. Doganay M, Demiraslan H. Refugees of the Syrian civil war: impact on reemerging infections, health services, and biosecurity in Turkey. Health security. 2016;14(4):220-5.
2. Guendelman S, Schauffler HH, Pearl M. Unfriendly shores: how immigrant children fare in the US health system. Health Affairs. 2001;20(1):257-66.
3. Crockett M. New faces from faraway places: Immigrant child health in Canada. Paediatrics & child health. 2005;10(5):277-81.
4. Sanmartin C, Ross N. Experiencing difficulties accessing first-contact health services in Canada: Canadians without regular doctors and recent immigrants have difficulties accessing first-contact healthcare services. Reports of difficulties in accessing care vary by age, sex and region. Healthcare Policy. 2006;1(2):103-19.
5. Pottie K, Greenaway C, Feightner J, Welch V, Swinkels H, Rashid M, et al. Evidence-based clinical guidelines for immigrants and refugees. Cmaj. 2011;183(12):E824-E925.
6. Doocy S, Lyles E, Roberton T, Akhu-Zaheya L, Oweis A, Burnham G. Prevalence and care-seeking for chronic diseases among Syrian refugees in Jordan. BMC Public Health. 2015;15(1):1097-2106.
7. Bailey C, Kodack M. Patient adherence to medication requirements for therapy of type 2 diabetes. International journal of clinical practice. 2011;65(3):314-22.
8. Basheti IA, El Hait SS, Qunaibi EA, Aburuz S, Bulatova N. Associations between patient factors and medication adherence: A Jordanian experience. Pharmacy Practice (Granada). 2016;14(1):1-7.
9. Ascione FJ, Kirscht JP, Shimp LA. An assessment of different components of patient medication knowledge. Medical care. 1986:1018-28.
10. Williford SL, Johnson DF. Impact of pharmacist counseling on medication knowledge and compliance. Military medicine. 1995;160(11):561-4.
11. Mahdikhani S, Dabaghzadeh F. Benefits of Pharmacist's Participation on Hospitalist Team. Acta Medica Iranica. 2016;54(2):140-5.
12. Ramalho de Oliveira D, Brummel AR, Miller DB. Medication therapy management: 10 years of experience in a large integrated health care system. Journal of Managed Care Pharmacy. 2010;16(3):185-95.
13. Castelino RL, Bajorek BV, Chen TF. Are interventions recommended by pharmacists during Home Medicines Review evidence-based? Journal of evaluation in clinical practice. 2011;17(1):104-10.
14. Bluml BM. Definition of medication therapy management: development of professionwide consensus. Journal of the American Pharmacists Association. 2005;45(5):566-72.
15. Basheti IA, Al-Qudah RA, Obeidat NM, Bulatova NR. Home medication management review in outpatients with chronic diseases in Jordan: a randomized control trial. International journal of clinical pharmacy. 2016;38(2):404-13.
16. Saleem F, Hassali MA, Shafie AA, Ul Haq N, Farooqui M, Aljadhay H, et al. Pharmacist intervention in improving hypertension-related knowledge, treatment medication adherence and health-related quality of life: a non-clinical randomized controlled trial. Health Expectations. 2015;18(5):1270-81.
17. Sirin SR, Rogers-Sirin L. The educational and mental health needs of Syrian refugee children: Migration Policy Institute Washington, DC; 2015, Acced by: http://wellnessresources.pbworks.com/w/file/fetch/117239565/Education%20and%20Mental%20Health%20Needs%20of%20Syrian%20Refugee%20Children.pdf.

18. Council APA. Guidelines for medication management in residential aged care facilities. Accessed by: https://www.health.gov.au/sites/default/files/documents/2020/02/guiding-principles-for-medication-management-in-residential-aged-care-facilities.pdf. 2007:1-98.
19. Freeman CR, Cottrell WN, Kyle G, Williams ID, Nissen L. An evaluation of medication review reports across different settings. International journal of clinical pharmacy. 2013;35(1):5-13.
20. Al Alawneh M, Nuaimi N, Basheti IA. Pharmacists in humanitarian crisis settings: Assessing the impact of pharmacist-delivered home medication management review service to Syrian refugees in Jordan. Research in social & administrative pharmacy : RSAP. 2019;15(2):164-72.
21. AbuRuz SM, Bulatova NR, Yousef A-MM, Al-Ghazawi MA, Alawwa IA, Al-Saleh A. Comprehensive assessment of treatment related problems in hospitalized medicine patients in Jordan. International journal of clinical pharmacy. 2011;33(3):501-11.
22. Al-Qudah RA, Bulatova NR, Obeidatc NM, Basheti IA. Impact of home medication management review on medication adherence among Jordanian patients. Journal of Pharmaceutical Health Services Research. 2018;[Accepted].
23. Coutts A, Fouad FM. Response to Syria's health crisis—poor and uncoordinated. The Lancet. 2013;381(9885):2242-3.
24. El-Khatib Z, Scales D, Vearey J, Forsberg BC. Syrian refugees, between rocky crisis in Syria and hard inaccessibility to healthcare services in Lebanon and Jordan. Conflict and health. 2013;7(1):18.
25. Abughosh SM, Wang X, Serna O, Henges C, Masilamani S, James Essien E, et al. A pharmacist telephone intervention to identify adherence barriers and improve adherence among nonadherent patients with comorbid hypertension and diabetes in a Medicare Advantage Plan. Journal of managed care & specialty pharmacy. 2016;22(1):63-73.
26. Mossialos E, Courtin E, Naci H, Benrimoj S, Bouvy M, Farris K, et al. From “retailers” to health care providers: transforming the role of community pharmacists in chronic disease management. Health Policy. 2015;119(5):628-39.
27. Lam WY, Fresco P. Medication adherence measures: an overview. BioMed research international. 2015:1-12.
28. Krousel-Wood M, Elizabeth H, Joyce C, Rachael R, Dornelles A, Webber LS, et al. Differences in cardiovascular disease risk when antihypertensive medication adherence is assessed by pharmacy fill versus self-report: the Cohort Study of Medication Adherence among Older Adults (CoSMO). Journal of hypertension. 2015;33(2):412-21.
29. Pellegrino AN, Martin MT, Tilton JJ, Touchette DR. Medication therapy management services. Drugs. 2009;69(4):393-406.
30. Brummel A, Carlson AM. Comprehensive medication management and medication adherence for chronic conditions. Journal of managed care & specialty pharmacy. 2016;22(1):56-62.
31. Tan X, Patel I, Chang J. Review of the four item Morisky medication adherence scale (MMAS-4) and eight item Morisky medication adherence scale (MMAS-8). INNOVATIONS in pharmacy. 2014;5(3):5-35.
32. Al-Eidan F, McElnay J, Scott M, McConnell J. Management of Helicobacter pylori eradication–the influence of structured counselling and follow-up. British journal of clinical pharmacology. 2002;53(2):163-71.
33. Jarab AS, Alqudah SG, Mukattash TL, Shattat G, Al-Qirim T. Randomized controlled trial of clinical pharmacy management of patients with type 2 diabetes in an outpatient diabetes clinic in Jordan. Journal of Managed Care Pharmacy. 2012;18(7):516-26.
34. Chow EP, Hassali MA, Saleem F, Aljadhey H. Effects of pharmacist-led patient education on diabetes-related knowledge and medication adherence: a home-based study. Health Education Journal. 2016;75(4):421-33.
35. Chisholm-Burns MA, Lee JK, Spivey CA, Slack M, Herrier RN, Hall-Lipsy E, et al. US pharmacists' effect as team members on patient care: systematic review and meta-analyses. Medical care. 2010:923-33.

36. World Health Organization. Seventieth World Health Assembly. Provisional agenda item 13.1. Human resources for health and implementation of the outcomes of the United Nations' High Level Commission on Health Employment and Economic Growth. World Health Assembly Journal Available at 18 http://appswhoint/gb/ebwha/pdf_files/WHA70'/A70_18-enpdf?ua=1 Accessed 4 February 2018. 2017.
37. World Health Organization. High-Level Commission on Health Employment and Economic Growth. Working for health and growth: investing in the health workforce. Report of the High-Level Commission on Health Employment and Economic Growth. Found at http://apps.who.int/iris/bitstream/10665/250047/1/9789241511308-eng.pdf. Accesssed on 4 February 2018. . 2016.

1. Doganay M, Demiraslan H. Refugees of the Syrian civil war: impact on reemerging infections, health services, and biosecurity in Turkey. Health security. 2016;14(4):220-5.
2. Guendelman S, Schauffler HH, Pearl M. Unfriendly shores: how immigrant children fare in the US health system. Health Affairs. 2001;20(1):257-66.
3. Crockett M. New faces from faraway places: Immigrant child health in Canada. Paediatrics & child health. 2005;10(5):277-81.
4. Sanmartin C, Ross N. Experiencing difficulties accessing first-contact health services in Canada: Canadians without regular doctors and recent immigrants have difficulties accessing first-contact healthcare services. Reports of difficulties in accessing care vary by age, sex and region. Healthcare Policy. 2006;1(2):103-19.
5. Pottie K, Greenaway C, Feightner J, Welch V, Swinkels H, Rashid M, et al. Evidence-based clinical guidelines for immigrants and refugees. Cmaj. 2011;183(12):E824-E925.
6. Doocy S, Lyles E, Roberton T, Akhu-Zaheya L, Oweis A, Burnham G. Prevalence and care-seeking for chronic diseases among Syrian refugees in Jordan. BMC Public Health. 2015;15(1):1097-2106.
7. Bailey C, Kodack M. Patient adherence to medication requirements for therapy of type 2 diabetes. International journal of clinical practice. 2011;65(3):314-22.
8. Basheti IA, El Hait SS, Qunaibi EA, Aburuz S, Bulatova N. Associations between patient factors and medication adherence: A Jordanian experience. Pharmacy Practice (Granada). 2016;14(1):1-7.
9. Ascione FJ, Kirscht JP, Shimp LA. An assessment of different components of patient medication knowledge. Medical care. 1986:1018-28.
10. Williford SL, Johnson DF. Impact of pharmacist counseling on medication knowledge and compliance. Military medicine. 1995;160(11):561-4.
11. Mahdikhani S, Dabaghzadeh F. Benefits of Pharmacist's Participation on Hospitalist Team. Acta Medica Iranica. 2016;54(2):140-5.
12. Ramalho de Oliveira D, Brummel AR, Miller DB. Medication therapy management: 10 years of experience in a large integrated health care system. Journal of Managed Care Pharmacy. 2010;16(3):185-95.
13. Castelino RL, Bajorek BV, Chen TF. Are interventions recommended by pharmacists during Home Medicines Review evidence-based? Journal of evaluation in clinical practice. 2011;17(1):104-10.
14. Bluml BM. Definition of medication therapy management: development of professionwide consensus. Journal of the American Pharmacists Association. 2005;45(5):566-72.
15. Basheti IA, Al-Qudah RA, Obeidat NM, Bulatova NR. Home medication management review in outpatients with chronic diseases in Jordan: a randomized control trial. International journal of clinical pharmacy. 2016;38(2):404-13.
16. Saleem F, Hassali MA, Shafie AA, Ul Haq N, Farooqui M, Aljadhay H, et al. Pharmacist intervention in improving hypertension-related knowledge, treatment medication adherence and health-related quality of life: a non-clinical randomized controlled trial. Health Expectations. 2015;18(5):1270-81.

17. Sirin SR, Rogers-Sirin L. The educational and mental health needs of Syrian refugee children: Migration Policy Institute Washington, DC; 2015, Acced by: http://wellnessresources.pbworks.com/w/file/fetch/117239565/Education%20and%20Mental%20Health%20Needs%20of%20Syrian%20Refugee%20Children.pdf.
18. Council APA. Guidelines for medication management in residential aged care facilities. Accessed by: https://www.health.gov.au/sites/default/files/documents/2020/02/guiding-principles-for-medication-management-in-residential-aged-care-facilities.pdf. 2007:1-98.
19. Freeman CR, Cottrell WN, Kyle G, Williams ID, Nissen L. An evaluation of medication review reports across different settings. International journal of clinical pharmacy. 2013;35(1):5-13.
20. Al Alawneh M, Nuaimi N, Basheti IA. Pharmacists in humanitarian crisis settings: Assessing the impact of pharmacist-delivered home medication management review service to Syrian refugees in Jordan. Research in social & administrative pharmacy : RSAP. 2019;15(2):164-72.
21. AbuRuz SM, Bulatova NR, Yousef A-MM, Al-Ghazawi MA, Alawwa IA, Al-Saleh A. Comprehensive assessment of treatment related problems in hospitalized medicine patients in Jordan. International journal of clinical pharmacy. 2011;33(3):501-11.
22. Al-Qudah RA, Bulatova NR, Obeidatc NM, Basheti IA. Impact of home medication management review on medication adherence among Jordanian patients. Journal of Pharmaceutical Health Services Research. 2018;[Accepted].
23. Coutts A, Fouad FM. Response to Syria's health crisis—poor and uncoordinated. The Lancet. 2013;381(9885):2242-3.
24. El-Khatib Z, Scales D, Vearey J, Forsberg BC. Syrian refugees, between rocky crisis in Syria and hard inaccessibility to healthcare services in Lebanon and Jordan. Conflict and health. 2013;7(1):18.
25. Abughosh SM, Wang X, Serna O, Henges C, Masilamani S, James Essien E, et al. A pharmacist telephone intervention to identify adherence barriers and improve adherence among nonadherent patients with comorbid hypertension and diabetes in a Medicare Advantage Plan. Journal of managed care & specialty pharmacy. 2016;22(1):63-73.
26. Mossialos E, Courtin E, Naci H, Benrimoj S, Bouvy M, Farris K, et al. From “retailers” to health care providers: transforming the role of community pharmacists in chronic disease management. Health Policy. 2015;119(5):628-39.
27. Lam WY, Fresco P. Medication adherence measures: an overview. BioMed research international. 2015:1-12.
28. Krousel-Wood M, Elizabeth H, Joyce C, Rachael R, Dornelles A, Webber LS, et al. Differences in cardiovascular disease risk when antihypertensive medication adherence is assessed by pharmacy fill versus self-report: the Cohort Study of Medication Adherence among Older Adults (CoSMO). Journal of hypertension. 2015;33(2):412-21.
29. Pellegrino AN, Martin MT, Tilton JJ, Touchette DR. Medication therapy management services. Drugs. 2009;69(4):393-406.
30. Brummel A, Carlson AM. Comprehensive medication management and medication adherence for chronic conditions. Journal of managed care & specialty pharmacy. 2016;22(1):56-62.
31. Tan X, Patel I, Chang J. Review of the four item Morisky medication adherence scale (MMAS-4) and eight item Morisky medication adherence scale (MMAS-8). INNOVATIONS in pharmacy. 2014;5(3):5-35.
32. Al-Eidan F, McElnay J, Scott M, McConnell J. Management of Helicobacter pylori eradication–the influence of structured counselling and follow-up. British journal of clinical pharmacology. 2002;53(2):163-71.
33. Jarab AS, Alqudah SG, Mukattash TL, Shattat G, Al-Qirim T. Randomized controlled trial of clinical pharmacy management of patients with type 2 diabetes in an outpatient diabetes clinic in Jordan. Journal of Managed Care Pharmacy. 2012;18(7):516-26.
34. Chow EP, Hassali MA, Saleem F, Aljadhey H. Effects of pharmacist-led patient education on diabetes-related knowledge and medication adherence: a home-based study. Health Education Journal. 2016;75(4):421-33.

35. Chisholm-Burns MA, Lee JK, Spivey CA, Slack M, Herrier RN, Hall-Lipsy E, et al. US pharmacists' effect as team members on patient care: systematic review and meta-analyses. Medical care. 2010:923-33.
36. World Health Organization. Seventieth World Health Assembly. Provisional agenda item 13.1. Human resources for health and implementation of the outcomes of the United Nations' High Level Commission on Health Employment and Economic Growth. World Health Assembly Journal Available at 18 http://appswhoint/gb/ebwha/pdf_files/WHA70'/A70_18-enpdf?ua=1 Accessed 4 February 2018. 2017.
37. World Health Organization. High-Level Commission on Health Employment and Economic Growth. Working for health and growth: investing in the health workforce. Report of the High-Level Commission on Health Employment and Economic Growth. Found at http://apps.who.int/iris/bitstream/10665/250047/1/9789241511308-eng.pdf. Accesssed on 4 February 2018. . 2016.

1. Doganay M, Demiraslan H. Refugees of the Syrian civil war: impact on reemerging infections, health services, and biosecurity in Turkey. Health security. 2016;14(4):220-5.
2. Guendelman S, Schauffler HH, Pearl M. Unfriendly shores: how immigrant children fare in the US health system. Health Affairs. 2001;20(1):257-66.
3. Crockett M. New faces from faraway places: Immigrant child health in Canada. Paediatrics & child health. 2005;10(5):277-81.
4. Sanmartin C, Ross N. Experiencing difficulties accessing first-contact health services in Canada: Canadians without regular doctors and recent immigrants have difficulties accessing first-contact healthcare services. Reports of difficulties in accessing care vary by age, sex and region. Healthcare Policy. 2006;1(2):103.
5. Pottie K, Greenaway C, Feightner J, Welch V, Swinkels H, Rashid M, et al. Evidence-based clinical guidelines for immigrants and refugees. Cmaj. 2011;183(12):E824-E925.
6. Doocy S, Lyles E, Roberton T, Akhu-Zaheya L, Oweis A, Burnham G. Prevalence and care-seeking for chronic diseases among Syrian refugees in Jordan. BMC Public Health. 2015;15(1):1097-2106.
7. Bailey C, Kodack M. Patient adherence to medication requirements for therapy of type 2 diabetes. International journal of clinical practice. 2011;65(3):314-22.
8. Basheti IA, El Hait SS, Qunaibi EA, Aburuz S, Bulatova N. Associations between patient factors and medication adherence: A Jordanian experience. Pharmacy Practice (Granada). 2016;14(1):1-7.
9. Ascione FJ, Kirscht JP, Shimp LA. An assessment of different components of patient medication knowledge. Medical care. 1986:1018-28.
10. Williford SL, Johnson DF. Impact of pharmacist counseling on medication knowledge and compliance. Military medicine. 1995;160(11):561-4.
11. Mahdikhani S, Dabaghzadeh F. Benefits of Pharmacist's Participation on Hospitalist Team. Acta Medica Iranica. 2016;54(2):140-5.
12. Ramalho de Oliveira D, Brummel AR, Miller DB. Medication therapy management: 10 years of experience in a large integrated health care system. Journal of Managed Care Pharmacy. 2010;16(3):185-95.
13. Castelino RL, Bajorek BV, Chen TF. Are interventions recommended by pharmacists during Home Medicines Review evidence-based? Journal of evaluation in clinical practice. 2011;17(1):104-10.
14. Bluml BM. Definition of medication therapy management: development of professionwide consensus. Journal of the American Pharmacists Association. 2005;45(5):566-72.
15. Basheti IA, Al-Qudah RA, Obeidat NM, Bulatova NR. Home medication management review in outpatients with chronic diseases in Jordan: a randomized control trial. International journal of clinical pharmacy. 2016;38(2):404-13.
16. Saleem F, Hassali MA, Shafie AA, Ul Haq N, Farooqui M, Aljadhay H, et al. Pharmacist intervention in improving hypertension-related knowledge, treatment medication adherence and

health-related quality of life: a non-clinical randomized controlled trial. Health Expectations. 2015;18(5):1270-81.
17. Sirin SR, Rogers-Sirin L. The educational and mental health needs of Syrian refugee children: Migration Policy Institute Washington, DC; 2015.
18. Council APA. Guidelines for medication management in residential aged care facilities. Accessed by: https://www.health.gov.au/sites/default/files/documents/2020/02/guiding-principles-for-medication-management-in-residential-aged-care-facilities.pdf. 2007:1-98.
19. Freeman CR, Cottrell WN, Kyle G, Williams ID, Nissen L. An evaluation of medication review reports across different settings. International journal of clinical pharmacy. 2013;35(1):5-13.
20. AbuRuz SM, Bulatova NR, Yousef A-MM, Al-Ghazawi MA, Alawwa IA, Al-Saleh A. Comprehensive assessment of treatment related problems in hospitalized medicine patients in Jordan. International journal of clinical pharmacy. 2011;33(3):501-11.
21. Al-Qudah RA, Bulatova NR, Obeidatc NM, Basheti IA. Impact of home medication management review on medication adherence among Jordanian patients. Journal of Pharmaceutical Health Services Research. 2018;[Accepted].
22. Coutts A, Fouad FM. Response to Syria's health crisis—poor and uncoordinated. The Lancet. 2013;381(9885):2242-3.
23. El-Khatib Z, Scales D, Vearey J, Forsberg BC. Syrian refugees, between rocky crisis in Syria and hard inaccessibility to healthcare services in Lebanon and Jordan. Conflict and health. 2013;7(1):18.
24. Abughosh SM, Wang X, Serna O, Henges C, Masilamani S, James Essien E, et al. A pharmacist telephone intervention to identify adherence barriers and improve adherence among nonadherent patients with comorbid hypertension and diabetes in a Medicare Advantage Plan. Journal of managed care & specialty pharmacy. 2016;22(1):63-73.
25. Mossialos E, Courtin E, Naci H, Benrimoj S, Bouvy M, Farris K, et al. From “retailers” to health care providers: transforming the role of community pharmacists in chronic disease management. Health Policy. 2015;119(5):628-39.
26. Lam WY, Fresco P. Medication adherence measures: an overview. BioMed research international. 2015:1-12.
27. Krousel-Wood M, Elizabeth H, Joyce C, Rachael R, Dornelles A, Webber LS, et al. Differences in cardiovascular disease risk when antihypertensive medication adherence is assessed by pharmacy fill versus self-report: the Cohort Study of Medication Adherence among Older Adults (CoSMO). Journal of hypertension. 2015;33(2):412.
28. Pellegrino AN, Martin MT, Tilton JJ, Touchette DR. Medication therapy management services. Drugs. 2009;69(4):393-406.
29. Brummel A, Carlson AM. Comprehensive medication management and medication adherence for chronic conditions. Journal of managed care & specialty pharmacy. 2016;22(1):56-62.
30. Tan X, Patel I, Chang J. Review of the four item Morisky medication adherence scale (MMAS-4) and eight item Morisky medication adherence scale (MMAS-8). INNOVATIONS in pharmacy. 2014;5(3):5.
31. Al-Eidan F, McElnay J, Scott M, McConnell J. Management of Helicobacter pylori eradication–the influence of structured counselling and follow-up. British journal of clinical pharmacology. 2002;53(2):163-71.
32. Jarab AS, Alqudah SG, Mukattash TL, Shattat G, Al-Qirim T. Randomized controlled trial of clinical pharmacy management of patients with type 2 diabetes in an outpatient diabetes clinic in Jordan. Journal of Managed Care Pharmacy. 2012;18(7):516-26.
33. Chow EP, Hassali MA, Saleem F, Aljadhey H. Effects of pharmacist-led patient education on diabetes-related knowledge and medication adherence: a home-based study. Health Education Journal. 2016;75(4):421-33.
34. Chisholm-Burns MA, Lee JK, Spivey CA, Slack M, Herrier RN, Hall-Lipsy E, et al. US pharmacists' effect as team members on patient care: systematic review and meta-analyses. Medical care. 2010:923-33.

35. World Health Organization. Seventieth World Health Assembly. Provisional agenda item 13.1. Human resources for health and implementation of the outcomes of the United Nations’ High Level Commission on Health Employment and Economic Growth. World Health Assembly Journal Available at 18 http://appswhoint/gb/ebwha/pdf_files/WHA70’/A70_18-enpdf?ua=1 Accessed 4 February 2018. 2017.

36. World Health Organization. High-Level Commission on Health Employment and Economic Growth. Working for health and growth: investing in the health workforce. Report of the High-Level Commission on Health Employment and Economic Growth. Found at http://apps.who.int/iris/bitstream/10665/250047/1/9789241511308-eng.pdf. Accesssed on 4 February 2018. . 2016.

Figure Click here to access/download;Figure;Figure 1.TIF

Figure (1). Consort diagram showing patients' recruitment and retention during the study period.

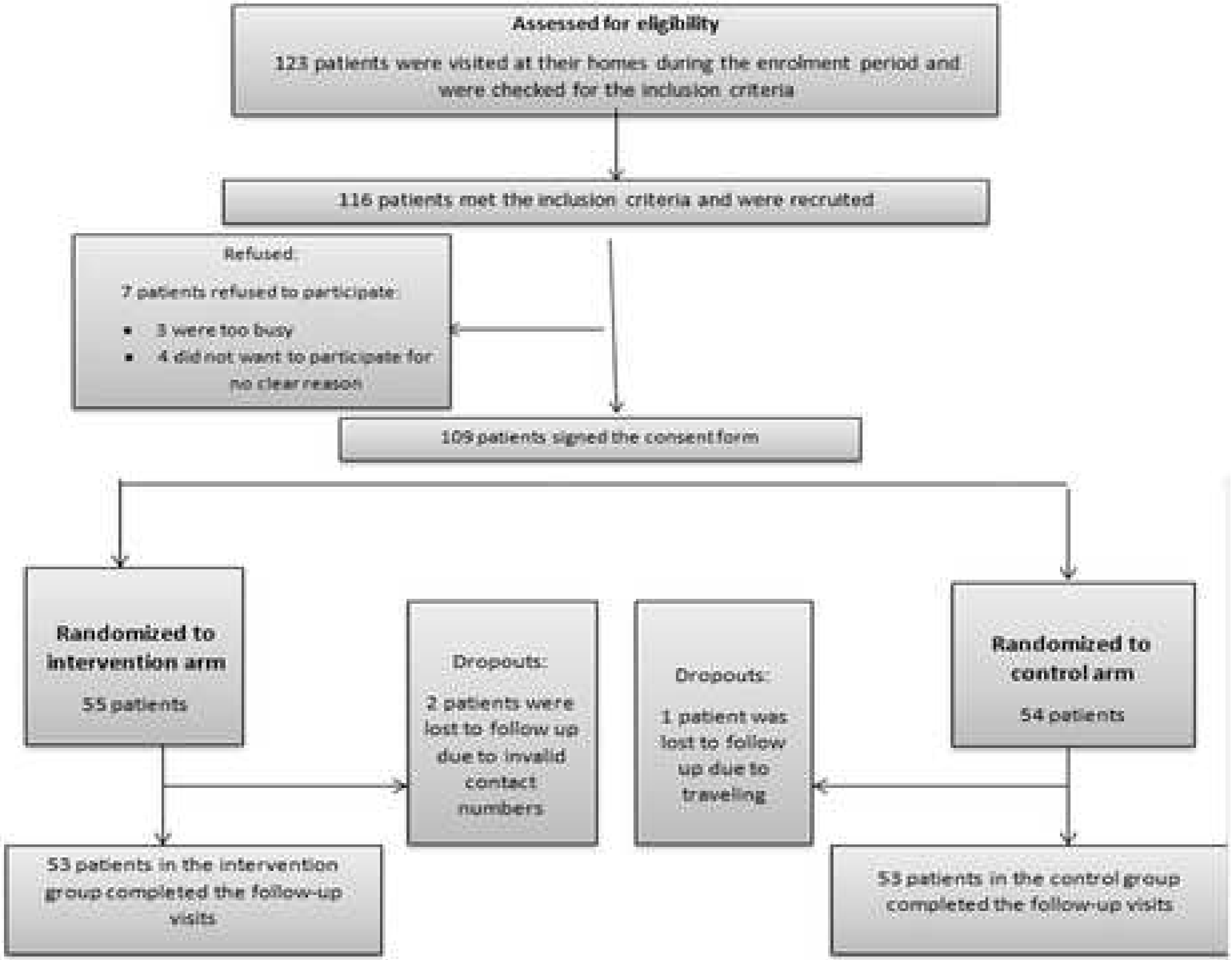

Figure (2). 'Adherence to medication' mean changes at baseline and follow-up

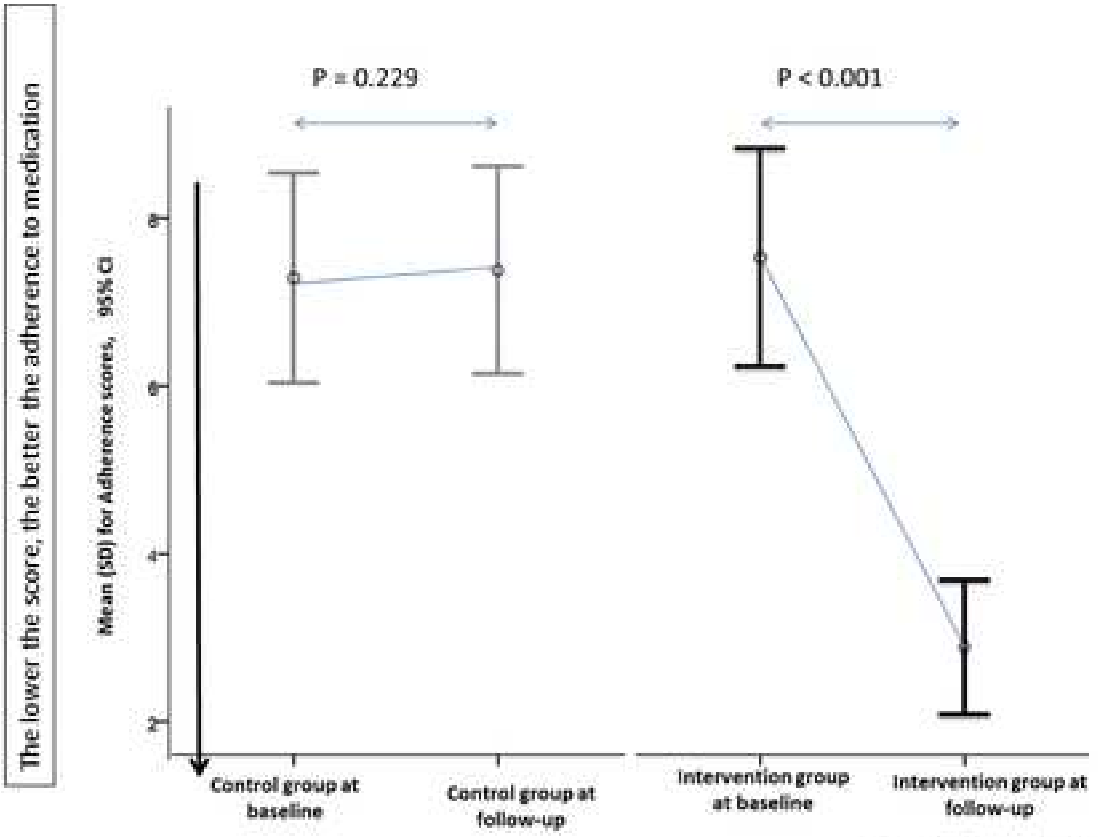

*P values by paired sample t-test

 Click here to access/download;Figure;Figure 3.TIF

Figure (3). The knowledge about drug therapy mean changes at baseline and follow-up

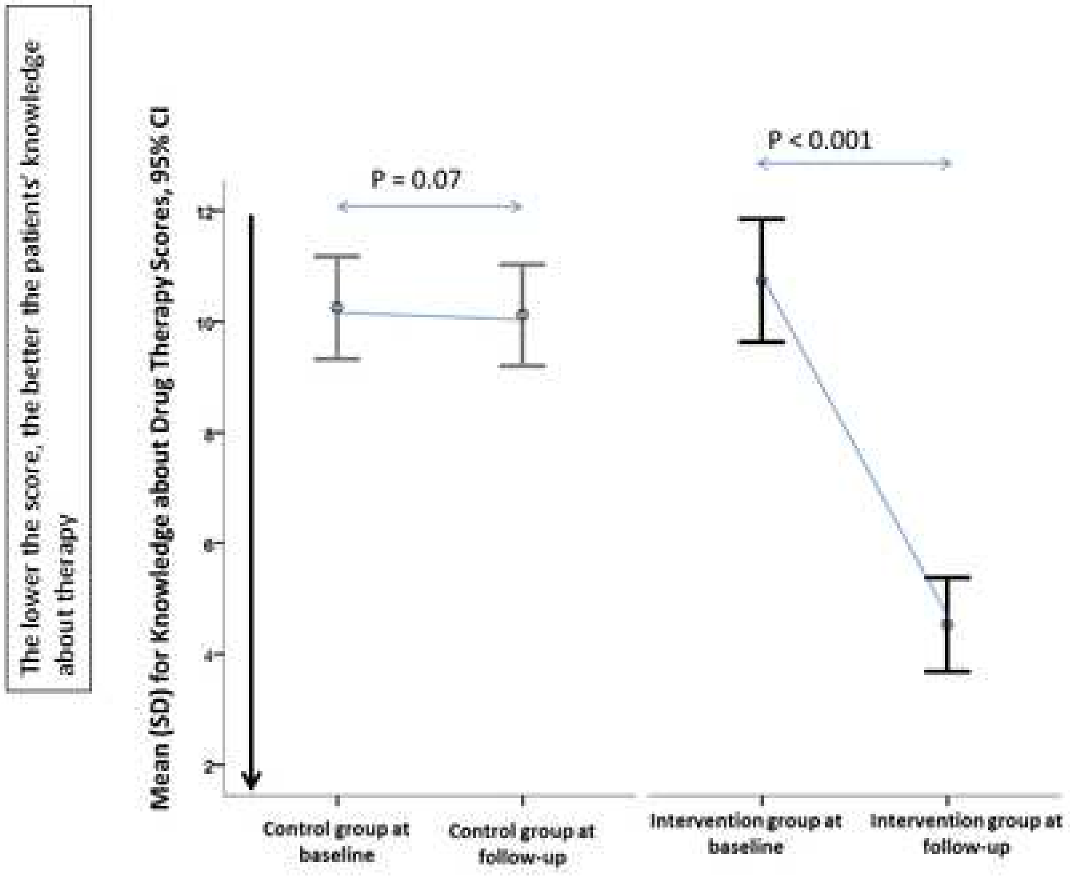

*P values by paired sample t-test

Click here to access/download
Supporting Information
IRB approval.docx

Study protocol

Click here to access/download

Supporting Information

OriginalStudyProtocol.Nov.docx
