# Supplementary material for: Improving Syrian refugees’ knowledge of medications and adherence following a randomized control trial assessing the effect of a medication management review service
Source: PLoS One. 2022 Oct 14;17(10):e0276304. doi: 10.1371/journal.pone.0276304 (PMC9565448; doi:10.1371/journal.pone.0276304)
Supplement: Attachment — Submitted filename: PlosOne 2021 = syrian refugees adherence.docx [file pone.0276304.s005.docx]

**Title**: Improving Syrian refugees’ knowledge of medications and adherence following a randomized control trial assessing the effect of a medication management review service

**Review comments:**

Line 3: Running title – remove capital R on refugees’

Line 44: what is chroexacnic? Should it be chronic

Line 48: I think knowledge of chronic medications may sound better than knowledge about chronic medications

Line 123: “following delivery of the service” may sound better

Line 129: I’m not sure what the point of registering the study four years after it was conducted. Can this be made clearer and not just say “delayed”.

Line 139: What impact does the reduced sample size have on confidence of findings? Please discuss in limitations section.

Line 151: Can you provide further detail about the clinical pharmacist. Was there just one for the study? How many years of experience do they have? Do they have additional qualifications?

Line 175: were recollected (not where)

Line 179: Was there any thought into the appropriateness of the data collection questionnaires for the refugees? I.e. did they have adequate health literacy to use these questionnaires?

Line 188: the scale appears to represent an ordinal scale, not a continuous scale. Can you please comment on the appropriateness of treating as a continuous scale?

Line 191: was the main cause of non-adherence an open question or did patients choose from a set list (as it seems from results in Table 2) – please clarify

Line 202: Was medication knowledge assessed per medication? Or as an aggregate measure? Also, was the patient-reported level of medication knowledge checked by the pharmacist?

Line 209: Were all continuous variables expressed as mean SD, was consideration given to parametric vs non-parametric distribution? The adherence questionnaires are typically skewed towards adherence.

Line 226: states 10 participants refused to continue however the numbers and figure 1 suggest this number was 7 participants. Please clarify.

Line 223: Was the mean number of medications and chronic conditions similar across groups. Can this be stated either in text or in table 1.

Line 238: Please keep consistent terminology throughout manuscript – active group or intervention group.

Results general: were there any patterns in the people who improved, e.g. based on baseline demographics. Was the service more effective for those that took more medications, had more complex regimens, had better education etc?

TRPs not mentioned in the results at all – yet they are listed in the protocol and abstract. Have they been omitted intentionally or accidently? Please add the results into the manuscript.

Line 278: improve lives is not supported by this study as quality of life was not reported. I note that quality of life is in your protocol – are those results going to be published separately? Please clarify.

Line 339: Further limitations to discuss include the subjective nature of your outcome measures, the fact that those that chose to participate may have been more receptive to advice/change, not meeting sample size etc

Line 342: why were recommendations approved/rejected not reported? What is the approval rate (an outcome specified in protocol)?

Discussion: consider discussing next steps, i.e. how could the intervention be further improved. 32% adherent is still very low and definitely not sufficient to reach adequate management of health. Consider comparing your intervention to others;

<https://www.cochranelibrary.com/cdsr/doi/10.1002/14651858.CD012419.pub2/full>

Reference: Not sure why reference list is duplicated. Please check.

Figure 2 &3: Is it possible to put the group baseline and follow-up scores on the graph too?
