# Supplementary material for: Improving Syrian refugees’ knowledge of medications and adherence following a randomized control trial assessing the effect of a medication management review service
Source: PLoS One. 2022 Oct 14;17(10):e0276304. doi: 10.1371/journal.pone.0276304 (PMC9565448; doi:10.1371/journal.pone.0276304)
Supplement: Attachment — Submitted filename: Response to reviewers.docx [file pone.0276304.s007.docx]

| Reviewer 1 | |
| --- | --- |
| Comment | Response |
| Reviewer #1: Thank you for addressing all comments. I have just one concern regarding the sample size. Since the study is underpowered, the results obtained might not be accurate. I will leave it up to the editor in chief to make the decision about accepting the paper or not. | Thank you for your valuable comment.  The recruited patients in the study were 116, and thus the recruitment was for 85% of the calculated sample size (138 patients) which hopefully could obtain the results with an enough accuracy. |
| Reviewer 2 | |
| Comment | Response |
| I can understand the purpose of the authors in doing that, but I can´t agree with them and so, with the redaction of the paragraph. In my opinion a “validated questionnaire” can´t be modified in any sense and so, the structure of the questions going from open-ended questions to close-ended questions, can´t be changed. This should be specified in the paper, perhaps including that “an ad hoc questionnaire was used based on a previously validated one, to help understanding to the respondents”. | The manuscript is modified as suggested (Please see Knowledge of chronic medications subheading, under the Methods) |
| Reviewer 3 | |
| Comment | Response |
| Qualitative answers are transformed into numeric values. Mapping the support of an ordered factor to the support of a numeric variable is in general not recommended, as it relies on a arbitrary choice. It is also not necessary, because generalized linear models (GLM) for ordered variables are available for analysis (also in SPSS, which is the software used in this paper). | **The indicated major issue is addressed by performing the following for Adherence scores and Knowledge scores:**  First: Generalized Linear Model Regression -Adherence – Preservice  We run the GLM (Ordinal logistic regression) via SPSS software for the Adherence questionnaire scores. The following is the SPSS Output for the performed test:  **Parameters Estimates Table:** the value of EXP(B) is **1.131 (**with P value = **0.716),** which does not suggest a decreasing probability with increasing values on independent variables.  Second: Correlation Analysis – Adherence - Preservice  The correlation between the adherence score and the MMR implementation was found to be statistically insignificant (P = **0.720,** Spearman’s rho), correlation value = 0.035 (poor correlation).  Third: Generalized Linear Model Regression -Adherence – Post service  We run the GLM (Ordinal logistic regression) via SPSS software for the Adherence questionnaire scores. The followings are the SPSS Output summery for the performed test:  **Parameters Estimates Table:** the value of EXP(B) is **0.134 (**with P value of **˂ 0.001),** which suggest a decreasing probability with increasing values on independent variables. This means that the scores of adherence decrease with the implementation of the MMR service (in other words: better adherence by patients upon providing MMR service by a factor of **0.134**).  Fourth: Correlation Analysis – Adherence - Post service  The correlation between the adherence score and the MMR implementation was found to be statistically significant (P **˂ 0.001,** Spearman’s rho), correlation value = 0.506 (good correlation).  Fifth: Generalized Linear Model Regression – Knowledge – Preservice  We run the GLM (Ordinal logistic regression) via SPSS software for the Knowledge questionnaire scores. The following is the SPSS Output for the performed test:  **Parameters Estimates Table:** the value of EXP(B) is 1.104 **(**with P value = 0.769**),** which does not suggest a decreasing probability with increasing values on independent variables.  Sixth: Correlation Analysis – Knowledge – Preservice  The correlation between the knowledge score and the MMR implementation was found to be statistically insignificant (P = 0.772**,** Spearman’s rho), correlation value = 0.028 (poor correlation).  Seventh: Generalized Linear Model Regression – Knowledge – Post service  We run the GLM (Ordinal logistic regression) via SPSS software for the Knowledge questionnaire scores. The followings are the SPSS Output summery for the performed test:  **Parameters Estimates Table:** the value of EXP(B) is **0.054 (**with P value of **˂ .001),** which suggest a decreasing probability with increasing values on independent variables. This means that the scores of knowledge decrease with the implementation of the MMR service (in other words: patients have better knowledge about their medications upon providing MMR service by a factor of **0.054**).  Eighth: Correlation Analysis – Knowledge – Post service  The correlation between the knowledge score and the MMR implementation was found to be statistically significant (P **˂ 0.001,** Spearman’s rho). correlation value = 0.671 (good correlation). |
| The response variable are obtained by summing scores derived from different questions. Summing heterogeneous scores that reflect different dimensions of adherence and knowledge make the interpretation of the response variable rather difficult: for instance, two subjects with different patterns of answers could obtain the same score. This is tolerable when the items are exchangeable. Is it the case here? If it is not, then the response variable should be treated as a multivariate response. An item response model is the state-of-the art method to analyze multiple items in a questionnaire. I don't know whether SPSS includes these models, but they are available in many software packages such as R and SAS. | The different items (dimensions) in both questionnaires are exchangeable. For example: the patients were considered under-adherent if they scored 1.0 or more in the total, regardless of the dimensions which they failed to achieve in the **eight-item Morisky Medication Adherence**.  Similarly, patients were considered to lacking the knowledge if they scored 1.0 or more in the total, regardless of the dimensions which they failed to achieve in the **Knowledge about Drug Therapy Questionnaire.** |
| Variable adherence is not a continuous variable: it is a discrete variable with range 0-32. Perhaps (not clear from text, see specific point 2) variable knowledge is discrete too. The t-test is therefore not fully appropriate in this context. Generalized linear mixed models (available in SPSS, which is the software used in the paper) provide the state-of-art strategy to compare discrete responses in a longitudinal setting. | The Generalized Linear Model (ordinal logistic regression) was performed instead of t-test, as suggested. The manuscript was modified accordingly. |
