# Supplementary material for: Improving Syrian refugees’ knowledge of medications and adherence following a randomized control trial assessing the effect of a medication management review service
Source: PLoS One. 2022 Oct 14;17(10):e0276304. doi: 10.1371/journal.pone.0276304 (PMC9565448; doi:10.1371/journal.pone.0276304)
Supplement: Attachment — Submitted filename: Response to reviewers.docx [file pone.0276304.s008.docx]

| The Editor | |
| --- | --- |
| Comment | Response |
| The concerns about the sample size should be further addressed in the Limitations and the conclusions should be interpreted with this in mind. | Thank you for your comment. To further address the reduced sample size concern, the following two modifications were done:   - In the **Limitation** section, the sentence was changed from *‘’The pre-specified sample size for the study was not achieved due to time constraints. This reduction in sample size and the subsequent increase in the margin of errors can affect the confidence level of the study’’* to*‘’* *The pre-specified sample size for the study was not achieved due to time constraints, to the difficulty that researcher faced in reaching refugees’ houses to complete the home visits at baseline and follow-up, and to the entire challenging nature of the study (*[*deplorable living conditions*](https://www.powerthesaurus.org/deplorable_living_conditions/synonyms) *which were seen throughout the whole data collection process). It is plausible that this reduction in sample size and the subsequent increase in the margin of errors can affect the confidence level and thus the results obtained from the study’’* - In the **Conclusion** section, the sentence was changed from ‘’*significant improvements in adherence and knowledge to treatment was reached as a result of the MMR service provided by the clinical pharmacist’’* to *‘’* *Despite that the recruitment was for 85% of the calculated sample size, the findings do nevertheless suggest that the significant improvements in adherence and knowledge to treatment was reached as a result of the MMR service provided by the clinical pharmacist’’* |
| Reviewer 3 | |
| Comment | Response |
| In my opinion, the key word “SDG 3” should be removed. | The indicated keyword is removed, as suggested. |
| In the Results section it is said that “the outcomes regarding the impact of the MMR on the TRPs’ type and frequency are reported elsewhere [28]” (Line 272). However, in the abstract some data on these results are included (At follow-up, a significant decrease in the number of TRPs for refugees in the intervention group was found ((from 600 to 182, P <0.001), but not for control group (number stayed at 541 TRPs, P= 0.116) (Line 60). I think that this information should, also, be removed from the Abstract | The indicated paragraph is removed from the Abstract, as suggested. |
