# Supplementary material for: Improving Syrian refugees’ knowledge of medications and adherence following a randomized control trial assessing the effect of a medication management review service
Source: PLoS One. 2022 Oct 14;17(10):e0276304. doi: 10.1371/journal.pone.0276304 (PMC9565448; doi:10.1371/journal.pone.0276304)
Supplement: Attachment — Submitted filename: Response to reviewers.docx [file pone.0276304.s009.docx]

| The Associate Editor Comment | |
| --- | --- |
| Comment | Response |
| The reviewers have not raised any major concerns concerning your manuscript. However during our in house checks we noticed you have used the 8 point Morisky Medication Adherence Scale. The authors of this scale patented the scale and it can only be used under license. Notably also we note you have included in your text several of the questions from the scale This text is copyrighted and can not be published. Before we can go forward with your manuscript these copyright issues need to be resolved. | The Aburuz et al adherence to medication questionnaire (appendix 1) which was used in the study is an updated version of Morisky et al questionnaire. One that fits our population. This updated, validated version [1] was used by many previous studies, and by us specifically, for the purpose of adherence evaluation among different populations [2-7]. No issues where raised by the morisky group regarding our use of the updated questionnaire. To further clarify this point so that no issues get raised, changes have been made in the manuscript accordingly. |

Appendix 1: Adherence to Medication Questionnaire

**الناس عادة يواجهون عدة مشاكل أثناء تناولهم لأدويتهم الموصوفة لتعددها واختلاف اوقاتها وأشكالها. نود أن نسألك بعض الأسئلة عن طبيعة استخدامك لدوائك.**

| **دائما** | **عادة** | **أحيانا** | **نادرا** | **إطلاقا** | **الأسئلة** |  |
| --- | --- | --- | --- | --- | --- | --- |
|  |  |  |  |  | **هل تنسى تناول دوائك؟** | **1** |
|  |  |  |  |  | **هل تتوقف عن تناول دوائك من وقت لآخر؟** | **2** |
|  |  |  |  |  | **هل تتوقف عن تناول دوائك عندما تشعر بتحسن؟** | **3** |
|  |  |  |  |  | **هل تتوقف عن تناول دوائك إذا ساءت حالتك بعد أخذ الدواء؟** | **4** |
|  |  |  |  |  | **هل تتوقف عن تناول دوائك إذا حصلت لك مضاعفات تعتقد انها ناتجة عن استخدام الدواء؟** | **5** |
|  |  |  |  |  | **هل تلتزم بنصائح الطبيب الصيدلاني فيما يتعلق بغذائك,ممارسة الرياضة,التدخين,الخ..؟** | **6** |
|  |  |  |  |  | **كم مرة أسبوعيا لا تتناول دوائك؟** | **7** |
|  |  |  |  |  | **ما هي اكثر الأسباب التي تعيقك عن أخذ دوائك:(أجب بنعم أو لا)**  **-السعر**  **-الوقت**  **-النسيان**  **-لا أحب الأدوية**  **-الدواء لا يعمل**  **-المضاعفات الناتجة عن الدواء**  **-كثرة عدد الأدوية** | **8** |

**احيانا: جرعة واحدة في الأسبوع**

**عادة: جرعتين في الأسبوع**

**دائما: أكثر من جرعتين في الأسبوع**

1.	AbuRuz SM, Bulatova NR, Yousef AM. Validation of a comprehensive classification tool for treatment-related problems. Pharmacy World and Science. 2006;28(4):222-32.

2.	AbuRuz SM, Bulatova NR, Yousef A-MM, Al-Ghazawi MA, Alawwa IA, Al-Saleh A. Comprehensive assessment of treatment related problems in hospitalized medicine patients in Jordan. International journal of clinical pharmacy. 2011;33(3):501-11.

3.	Basheti IA, Al-Qudah RA, Obeidat NM, Bulatova NR. Home medication management review in outpatients with chronic diseases in Jordan: a randomized control trial. International journal of clinical pharmacy. 2016;38(2):404-13.

4.	Basheti IA, Tadros OK, Aburuz S. Value of a community‐based medication management review service in Jordan: a prospective randomized controlled study. Pharmacotherapy: The Journal of Human Pharmacology and Drug Therapy. 2016;36(10):1075-86.

5.	Al-Qudah RA, Bulatova NR, Obeidat NM, Basheti IA. Impact of home medication management review on medication adherence among Jordanian patients. Journal of Pharmaceutical Health Services Research. 2018;9(3):227-35.

6.	Basheti IA, Rizik M, Bulatova NR. Home medication management review in outpatients with alarming health issues in Jordan: a randomized control trial. Journal of Pharmaceutical Health Services Research. 2018;9(2):91-100.

7.	AbuRuz S, Jaber D, Basheti I, Sadeq A, Arafat M, AlAhmad M, et al. Impact of pharmacist interventions on drug-related problems in general surgery patients: a randomised controlled trial. European Journal of Hospital Pharmacy. 2021;28(Suppl 2):s72-s8.
