# Supplementary material for: Improving Syrian refugees’ knowledge of medications and adherence following a randomized control trial assessing the effect of a medication management review service
Source: PLoS One. 2022 Oct 14;17(10):e0276304. doi: 10.1371/journal.pone.0276304 (PMC9565448; doi:10.1371/journal.pone.0276304)
Supplement: Attachment — Submitted filename: Response to Reviewers.docx [file pone.0276304.s010.docx]

**14 August 2022**

**Deputy Editor in Chief**

**Re: PLOS ONE Decision: Revision required [PONE-D-20-26688R4] - [EMID:5f1c5bc0cd3004f6]**

On the behalf of all the authors of the manuscript, I would like to thank you for providing the comments on the manuscript. We highly appreciate the comments and strongly believe that the comments would positively impact the quality and the scientific merit of the paper. We have revised the manuscript and incorporated appropriate changes to address the comments.

We have provided responses (see below) in response to reviewers’ comments.

Best regards,

Iman A. Basheti.; Corresponding author

Email: dr_iman@asu.edu.jo Phone number - +962 79904800

| Deputy Editor in Chief Comments | |
| --- | --- |
| Comment | Response |
| Please upload a completed CONSORT checklist as a supporting information file. | The complete CONSORT checklist is uploaded, as requested. |
| The information in the registry entry suggests that your trial was registered after patient recruitment began. PLOS ONE strongly encourages authors to register all trials before recruiting the first participant in a study. As per the journal’s editorial policy, please include in the Methods section of your paper: 1) your reasons for your delay in registering this study (after enrolment of participants started); 2) confirmation that all related trials are registered by stating: “The authors confirm that all ongoing and related trials for this drug/intervention are registered”. | 1) The reasons for the delayed registry are written under METHOD section. 2) The statement *‘’The authors confirm that all ongoing and related trials for this drug/intervention are registered’’* is added, as requested. |
